# Identification of a set of genes potentially responsible for resistance to ferroptosis in lung adenocarcinoma cancer stem cells

**DOI:** 10.1038/s41419-024-06667-w

**Published:** 2024-04-29

**Authors:** Francesca Ascenzi, Antonella Esposito, Sara Bruschini, Valentina Salvati, Claudia De Vitis, Valeria De Arcangelis, Giulia Ricci, Angiolina Catizione, Simona di Martino, Simonetta Buglioni, Massimiliano Bassi, Federico Venuta, Francesca De Nicola, Alice Massacci, Isabella Grassucci, Matteo Pallocca, Alberto Ricci, Maurizio Fanciulli, Gennaro Ciliberto, Rita Mancini

**Affiliations:** 1 Translational Oncology Research Unit, IRCCS Regina Elena National Cancer Institute, Rome, Italy; 2 Department of Clinical and Molecular Medicine, Sant’ Andrea Hospital-Sapienza University of Rome, Rome, Italy; 3 Department of Experimental and Clinical Medicine, Magna Graecia University of Catanzaro, Catanzaro, Italy; 4 SAFU Laboratory, IRCCS Regina Elena National Cancer Institute, Rome, Italy; 5 Preclinical Models and New Therapeutic Agents Unit, IRCCS Regina Elena National Cancer Institute, Rome, Italy; 6 Department of Experimental Medicine, Università Degli Studi Della Campania Luigi Vanvitelli, Naples, Italy; 7 Department of Anatomy, Histology, Forensic-Medicine and Orthopedics, Sapienza University of Rome, Rome, Italy; 8 Pathology Unit, IRCCS-Regina Elena National Cancer Institute, Rome, Italy; 9 Thoracic Surgery Unit, Sapienza University of Rome, Rome, Italy; 10 Biostatistics, Bioinformatics and Clinical Trial Center, IRCCS Regina Elena National Cancer Institute, Rome, Italy; 11 Institute of Experimental Endocrinology and Oncology, National Research Council, Naples, Italy; 12 Respiratory Unit, Sant’Andrea Hospital, Sapienza University of Rome, Rome, Italy; 13 Scientific Direction, IRCCS Regina Elena National Cancer Institute, Rome, Italy; 14 Present Address: Experimental Pharmacology Unit, Istituto Nazionale Tumori - IRCCS - Fondazione G. Pascale, Napoli, Italy

**Keywords:** Cancer stem cells, Non-small-cell lung cancer

## Abstract

Scientific literature supports the evidence that cancer stem cells (CSCs) retain inside low reactive oxygen species (ROS) levels and are, therefore, less susceptible to cell death, including ferroptosis, a type of cell death dependent on iron-driven lipid peroxidation. A collection of lung adenocarcinoma (LUAD) primary cell lines derived from malignant pleural effusions (MPEs) of patients was used to obtain 3D spheroids enriched for stem‐like properties. We observed that the ferroptosis inducer RSL3 triggered lipid peroxidation and cell death in LUAD cells when grown in 2D conditions; however, when grown in 3D conditions, all cell lines underwent a phenotypic switch, exhibiting substantial resistance to RSL3 and, therefore, protection against ferroptotic cell death. Interestingly, this phenomenon was reversed by disrupting 3D cells and growing them back in adherence, supporting the idea of CSCs plasticity, which holds that cancer cells have the dynamic ability to transition between a CSC state and a non-CSC state. Molecular analyses showed that ferroptosis resistance in 3D spheroids correlated with an increased expression of antioxidant genes and high levels of proteins involved in iron storage and export, indicating protection against oxidative stress and low availability of iron for the initiation of ferroptosis. Moreover, transcriptomic analyses highlighted a novel subset of genes commonly modulated in 3D spheroids and potentially capable of driving ferroptosis protection in LUAD-CSCs, thus allowing to better understand the mechanisms of CSC-mediated drug resistance in tumors.

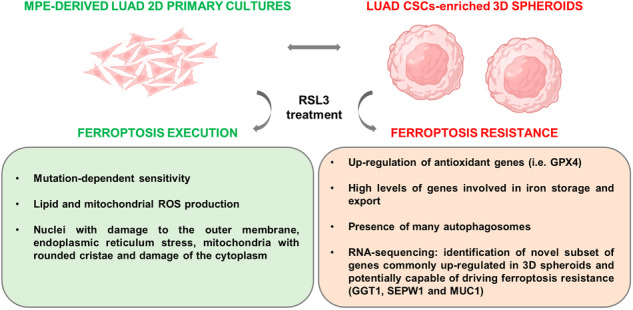

## Introduction

Lung cancer is the leading cause of cancer death worldwide and lung adenocarcinoma (LUAD) represents the most common histologic subtype, with about 40% of cases [1]. Currently, the most common treatment options for early stages include surgery and adjuvant therapies. Since the disease is often diagnosed at an advanced stage, half of patients die within the first year of diagnosis, and 5-year overall survival is 15 percent [1]. Therefore, understanding the molecular mechanisms of lung cancer is crucial for developing novel strategies aimed at managing and fighting this malignancy.

Cancer stem cells (CSCs) are believed to initiate and sustain tumor growth and are suggested to be responsible for drug resistance and cancer relapse. In fact, CSCs are highly dependent on antioxidant mechanisms for survival, resulting in less susceptibility to death by classical anticancer agents [2]. It has recently been demonstrated that many chemotherapy drugs induce ferroptosis, an intracellular iron-dependent form of cell death and that the dysregulation of ferroptosis often leads to chemotherapy resistance and treatment failure [3]. Ferroptosis occurs through the accumulation of lipid reactive oxygen species (ROS), and it is the result of an imbalance between the amount of lipid hydroperoxides in the cell and the levels of detoxifying enzymes [4, 5]. It has been recently suggested that the induction of ferroptosis may represent a new therapeutic strategy to target CSCs in different histological types of cancer, including lung cancer [6]. These results compelled us to conduct a more thorough investigation into this phenomenon in lung adenocarcinoma with the goal of identifying novel potential markers that might explain CSCs’ resistance to ferroptotic death.

It has been amply accepted that the condition of CSC is not fixed but can be reached when differentiated cancer cells are exposed to certain microenvironment-derived stimuli, such as hypoxia, cytokines, and epithelial-mesenchymal transition [7]. On the other hand, CSCs can be plastically driven towards a differentiated state [7]. In previous articles, we described a procedure for establishing tumor cell cultures from malignant pleural effusions (MPEs) obtained from LUAD patients [8, 9]. These cells can be grown in a serum‐free medium supplemented with growth factors and cultured in non-adherent conditions as three-dimensional (3D) spheroids [8–10]. We ascertained that spheroids are enhanced for stem-like characteristics, including the overexpression of Nanog, Oct4, and Sox2 markers, the increased aldehyde dehydrogenase (ALDH) activity, and the efficiency in tumor induction that mimic the same histological characteristics of the original human tumor when injected into recipient mice [8]. Moreover, we demonstrated that lung cancer 3D spheroids overexpress lipid metabolism-related genes, including stearoyl-CoA desaturase 1 (SCD1), the primary biosynthetic enzyme responsible for converting saturated fatty acids into monounsaturated fatty acids [11]. We also discovered that SCD1 was a crucial component for lung CSCs and that its inhibition reversed resistance to cisplatin treatment by working in concert with chemotherapy [11, 12]. Furthermore, a protective role of SCD1 against ferroptotic cell death has recently been demonstrated [13–16].

Since 3D spheroids incur profound changes in their lipid metabolism, in this article, we examined whether ferroptosis, that is solely dependent on lipid peroxidation levels, is compromised in this culture condition and linked to drug resistance in LUAD-CSCs. In particular, we selected a suitable collection of LUAD primary cultures, and we investigated the consequences of drug-induced ferroptosis in 3D and 2D in vitro models, dissecting the molecular and biochemical mechanisms behind the phenomenon of ferroptosis resistance. With this approach, we observed a profound molecular reprogramming between the two culture conditions, and we selected potential new genes capable of driving this process and predicting therapy response in LUAD. Although further studies are necessary, the research has nonetheless set the groundwork for understanding the mechanisms of drug resistance in LUAD.

## Results

### Establishment and characterization of primary tumor cells from lung adenocarcinoma cancer patients

Low passage primary cultures of tumor cells represent an excellent ex vivo model for translational research because they are believed to maintain the same features of resident cancer cells present in tumor lesions. Primary cell cultures of lung adenocarcinoma were obtained by isolating tumor cells from MPEs of patients, according to a specific isolation protocol previously described [8, 9]. In order to characterize primary cell cultures, they were subjected both to phenotypic and genotypic investigations. For the immunophenotypic characterization of the primary cultures, the expression levels of some specific markers were analysed by flow cytometry. In particular: epithelial cell adhesion molecule (EpCAM), expressed in epithelial cells and correlated with tumorigenesis [17]; Cytokeratin 7 (CK7), normally produced by lung epithelial cells and commonly used as a marker for diagnosis of lung tumors [18]; CD90 (alias Thymus cell antigen 1), a well-known marker of fibroblast [19, 20]; CD45, a ubiquitous cell surface marker of nucleated hematopoietic cells [21]. The LUAD primary cell cultures isolated exhibited a marked positivity for EpCAM and CK7/8 markers, assuring that they were constituted by cancer cells of epithelial origin with the same phenotypic features of the original tumor, as well as negligible expression of CD90 and CD45 markers, thus excluding the presence of fibroblasts and tumor-infiltrating immune cells (Table 1). Mutational profiling was performed by Next-Generation Sequencing (NGS), enabling the simultaneous detection of variants in 52 genes relevant to solid tumors. For this study, we selected four primary cultures (Table 1), all harboring mutations in the KRAS gene, accompanied or not by alterations in other genes, such as Keap1 and Tp53, known to be associated with protection from ferroptosis [14, 22].

**Table 1:** Phenotypic and genotypic characterization of MPE-derived primary cell cultures used in the study.

MPE-derived primary cell cultures	Patient sex/age	Patient diagnosis	Cytofluorimetric analysis of Cell cultures (% positive cells)				Mutational NGS analysis of Cell cultures
**BBIRE-T248**	M/74	Adenocarcinoma	EPCAM + 99.53%	CK7/8 + 94.90%	CD90 + 0.03%	CD45 + 0.03%	KRAS p.G12V (Level III/II), c.35 G > T, Exon2; VAF 86%
**PUC30**	F/58	Adenocarcinoma	EPCAM + 99.91%	CK7/8 + 99.15%	CD90 + 0.73%	CD45 + 0.92%	KRAS p.G12D (Level IV/IID), c.35 G > A, Exon2; VAF 33.5%
**PUC36**	F/62	Adenocarcinoma	EPCAM + 92.27%	CK7/8 + 90.45%	CD90 + 5.11%	CD45 + 0.30%	KRAS p.G12C (Level IIIA/IB), c.34 G > T, Exon2; VAF 99.5% TP53 p.R273H, c.818 G > A, Exon8; VAF 33% TP53 p.K132N, c.396 G > T, Exon5; VAF 69%
**PUC37**	M/66	Adenocarcinoma	EPCAM + 99.76%	CK7/8 + 93.23%	CD90 + 1.21%	CD45 + 1.53%	KRAS p.G12C (Level IIIA/IB), c.34 G > T, Exon2; VAF 66% TP53 p.W146*, c.438 G > A, Exon5; VAF 98% KEAP1 p.C368F, c.1103 G > T, Exon3; VAF 99%

### Lung adenocarcinoma CSCs are resistant to ferroptosis

**Fig. 1:**
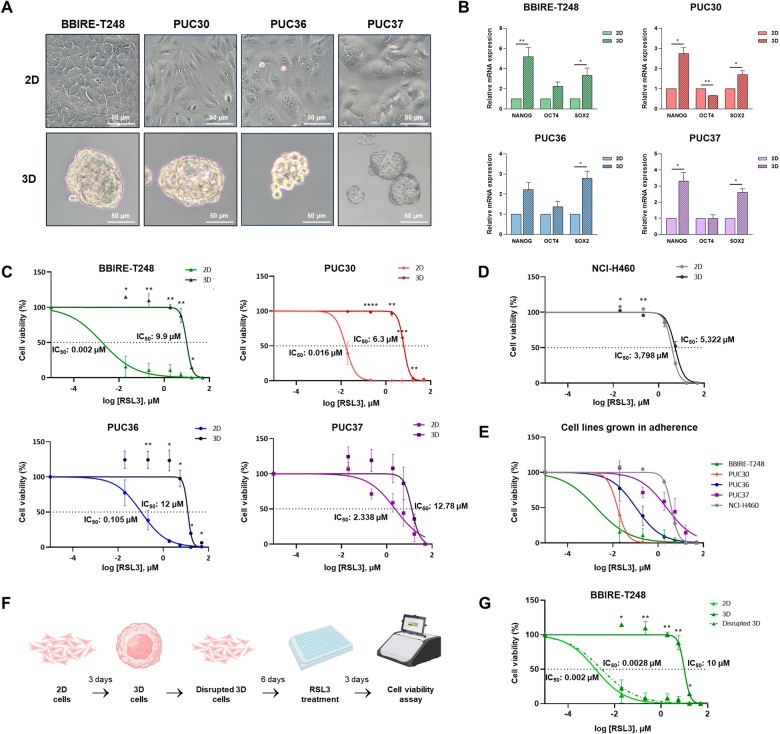
Lung adenocarcinoma CSCs are resistant to ferroptosis activator RSL3. **A** Representative images at 10x magnification of BBIRE-T248, PUC30, PUC36, and PUC37 primary cultures grown in adherent (2D) and in non-adherent (3D) conditions. The scale bar represents 50 μm. **B** Real-time PCR analyses of stemness-associated genes in primary cells cultured in 2D and 3D conditions. Data were representative of three independent experiments; values are expressed as mean ± SEM and are statistically significant if *p < 0.05 (paired Student’s *t*-test). **C**, **D** Dose-response curves of sensitivity to RSL3 at 72 h in BBIRE-T248, PUC30, PUC36, and PUC37 primary cultures (**C**) and in stable cell line NCI-H460 (**D**) grown in 2D and 3D conditions. **E** Comparison of dose-response curves of sensitivity to RSL3 in all cell lines grown in adherence (2D). **F** Workflow of the sphere disruption experiment and subsequent evaluation of sensitivity to RSL3 (Created with BioRender.com). **G** Dose-response curves of sensitivity to RSL3 at 72 h in BBIRE-T248 2D, 3D, and disrupted 3D cells. Data were representative of three independent experiments; values are expressed as mean ± SEM and are statistically significant if *p < 0.05 (Welch’s *t*-test) for the comparison between 2D and 3D (**C**, **D**) or for the comparison between 3D and disrupted 3D (**G**). The IC_50_ (half maximal inhibitory concentration) values are reported in the graphs.

Given the role of CSCs in cancer biology and resistance to therapy, targeting CSCs metabolism may serve as an important approach for future cancer treatments. Moreover, since lipid metabolism undergoes significant changes in CSCs [8, 11, 23], we investigated whether ferroptosis, a type of cell death strictly dependent upon the level of lipid peroxidation, is somehow impaired in CSCs and is associated with drug resistance in the 3D culture model. The four primary cell cultures selected for the study were thus grown in a serum‐free medium supplemented with growth factors and cultured in non-adherent conditions as three-dimensional spheroids to enrich for CSCs; in parallel, they were grown in adherence conditions. Figure 1A shows the morphological aspect of the four primary cultures grown in 2D and 3D contexts. In order to verify the enrichment of CSCs in 3D cultures, we evaluated by real-time PCR the expression of stem markers such as Nanog, Oct4, and Sox2 (Fig. 1B). Although not all markers show statistically significant upregulation in the spheroids, a general pattern of overexpression is observed. To evaluate the sensitivity of the two culture systems to ferroptotic cell death, we treated the four lung cancer primary cultures (BBIRE-T248, PUC30, PUC36, and PUC37) with RSL3, a ferroptosis activator with selectivity for tumor cells bearing oncogenic Ras, which acts by inhibiting the glutathione peroxidase 4 (GPX4). The data clearly show that cells grown in adherence were sensitive to the treatment with RSL3, while a high degree of resistance to this agent was observed in CSCs-enriched cultures (Fig. 1C). Based on the evidence that Stk11 and Keap1 co-mutations cooperate to promote protection from ferroptosis, independent from the Kras status [13], we also performed viability assays following RSL3 treatment in the NCI-H460 stable cell line that, having triple mutations in Kras-Stk11-Keap1 genes, could represent a positive control of ferroptosis resistance. As shown in Fig. 1D, the NCI-H460 cell line was confirmed to be highly resistant to RSL3, both in 2D and in 3D conditions. To note, the baseline sensitivity to RSL3 (relative to growth in adhesion) varied greatly across cancer cell lines, probably due to their mutational status (Kras, Tp53 and Keap1 mutations). In particular, the PUC37 cell culture, that harbors Kras-Keap1-Tp53 mutations, has a similar sensitivity to the NCI-H460 cell line, while the PUC30 cell culture, that harbors only Kras mutation, is similar to BBIRE-T248. Finally, the dose-response curve of PUC36 is in the middle of the graph, probably due to the additional mutation in Tp53 (Fig. 1E). In order to demonstrate the reproducibility, and, therefore the robustness of the phenomenon observed in primary cells, we also evaluated the sensitivity to RSL3 in three established cell lines grown in 2D and 3D conditions. Likewise, we observed that tumor cells grown as 3D spheroids are more resistant to RSL3-induced ferroptosis than their counterpart grown in adherent conditions (Fig. S1). Furthermore, by comparing the IC_50_ values of the three cell cultures grown in adhesion, we confirmed that the Tp53 and Keap1 mutations made the cells less sensitive to ferroptosis activators (Fig. S1 and Table S5).

These results led us to hypothesize that resistance to ferroptosis in lung CSCs may be linked to dynamic molecular and epigenetic changes occurring in cells when grown in 3D conditions. To verify this hypothesis, we disrupted the 3D spheroids of the BBIRE-T248 cell culture and grew cells in adherence for 6 days to then re-evaluate their sensitivity to RSL3 (Fig. 1F). 3D disrupted cells showed a sensitivity similar to 2D cells, supporting the concept of CSC plasticity in which cancer cells harbor the dynamic capacity to switch from a non-CSC state to a CSC state and then back to non-CSC, in parallel with their susceptibility to ferroptotic cell death (Fig. 1G).

### Lung adenocarcinoma CSCs resist RSL3-induced ferroptotic cell death by counteracting lipid and mitochondrial ROS production

**Fig. 2:**
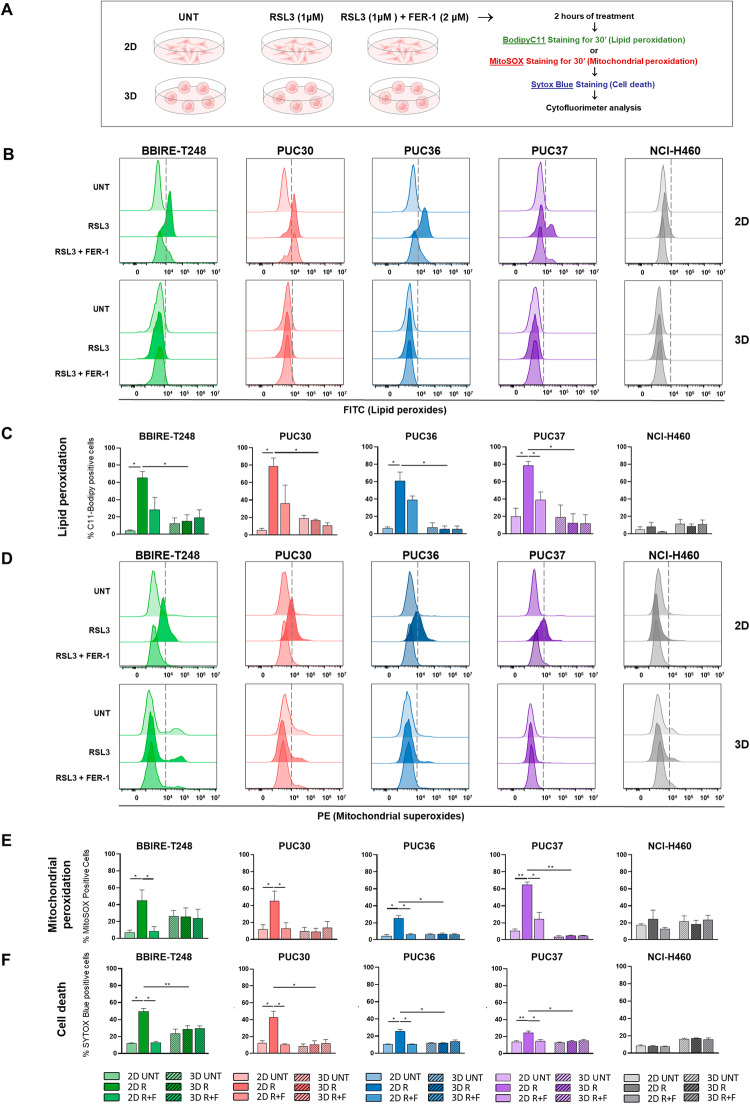
LUAD-CSCs resist to RSL3-induced ferroptosis by counteracting lipid and mitochondrial ROS production. **A** Workflow of measurement of lipid peroxidation, mitochondrial peroxidation, and cell death in primary and stable cell lines grown in 2D and 3D conditions following RSL3 and Ferrostatin-1 treatment (Created with BioRender.com). **B** Density curves of lipid peroxidation of a representative experiment of BBIRE-T248, PUC30, PUC36, PUC37, and NCI-H460 cell lines treated with DMSO (UNT), 1 μM RSL3 (RSL3), and 1 μM RSL3 + 2 μM Ferrostatin-1 (RSL3 + FER-1) for 2 h. **C** Histograms of three independent experiments of C11-BODIPY (581/591) staining to detect lipid peroxides. **D** Density curves of mitochondrial peroxidation of a representative experiment of BBIRE-T248, PUC30, PUC36, PUC37, and NCI-H460 cell lines treated with DMSO (UNT), 1 μM RSL3 (RSL3), and 1 μM RSL3 + 2 μM Ferrostatin-1 (RSL3 + FER-1) for 2 h. **E** Histograms of three independent experiments of MitoSOX staining to detect mitochondrial peroxides. **F** Histograms of three independent experiments of SYTOX Blue staining to quantify cell death (R: RSL3; F: Ferrostatin-1). All data were expressed as mean ± SEM and are statistically significant if *p < 0.05 and very significant if **p < 0.01 (paired Student’s *t*-test).

To understand the effect of RSL3 on the cell cultures described in Fig. 1, we investigated the phenomenon of lipid peroxidation, the core reaction of ferroptosis, in cells treated for 2 h with RSL3 and grown in 2D and in 3D conditions, respectively, for 72 h. Lipid peroxidation is caused by free radicals that attack unsaturated fatty acids of membranes producing lipid peroxides; in turn, lipid peroxides, as highly reactive compounds, propagate further generation of ROS and alter the assembly, composition, structure, and dynamics of lipid membranes, leading to membrane rupture and cell death [5]. We performed a flow cytometric analysis using C11-BODIPY (581/591) as reporter of lipid peroxidation and assessed the levels of lipid peroxides in samples treated with 1 μM RSL3 alone or in combination with 2 μM of Ferrostatin-1, a ferroptosis inhibitor with antioxidant capacity (Fig. 2A). In the same experimental setup, we also evaluated the percentage of dead cells (Fig. 2A). In Fig. 2B are reported the density curves of lipid peroxidation of a representative experiment for each cell line, while the histograms in Fig. 2C, F show the results of three independent experiments of measure of lipid peroxidation and cell death respectively. It is evident that 2D cell lines treated with RSL3 show increased levels of lipid peroxidation compared to the control but to a different extent, in line with their different sensitivity to cell death shown in the previous paragraph. Moreover, lipid peroxidation was rescued, at least in part, in cells cotreated with the antioxidant Ferrostatin-1, that acts via a reductive mechanism to prevent damage to membrane lipids, thereby inhibiting lipid peroxidation and cell death. Interestingly, the same cells grown in 3D conditions were completely resistant to lipid peroxidation, in perfect agreement with the lack of sensitivity to cell death induced by RSL3 (Figs. 1,2F). For the BBIRE-T248 cell line, we also confirmed these data visualizing lipid ROS by confocal microscopy. As shown in Fig. S2, 2D cells exhibit a stronger green fluorescence emission (the sign of lipid peroxidation) in RSL3-treated 2D cells compared to both the untreated 2D cells and the 2D cells treated with RSL3 and Ferrostatin-1 (Fig. S2A). On the contrary, BBIRE-T248 cells grown as spheroids do not show significant changes in fluorescence emission intensity between the conditions analysed (Fig. S2B).

Another class of ROS involved in ferroptosis is represented by the mitochondrial reactive oxygen species [24]. In order to investigate the oxidative stress by mitochondrial superoxides, we decided to perform flow cytometric analyses using the MitoSOX Red reagent, a fluorogenic dye specifically targeting mitochondria in live cells. In particular, oxidation of the MitoSOX Red reagent by mitochondrial superoxide produces red fluorescence indicative of cellular oxidative stress. We set up the experimental conditions culturing all cell lines previously investigated in 2D and 3D conditions and assessed the percentage of mitochondrial superoxides after 2 h of treatment with 1 μM RSL3 alone or in combination with 2 μM Ferrostatin-1 (Fig. 2A). As reported in the density curves of representative experiments in Fig. 2D, we observed that RSL3 treatment causes mitochondrial superoxide production in all 2D samples except for NCI-H460 cell line. Interestingly, at the basal level, cells grown in 3D conditions have a higher level of mitochondrial oxidative stress than cells grown in 2D conditions but treatment with RSL3 does not produce further mitochondrial superoxides production (Fig. 2E). For each cell line, three biological replicates were performed and the percentages of positive cells for MitoSOX Red dye are reported in the histograms in Fig. 2E. In all cases, the co-treatment with Ferrostatin-1 rescues the percentage of mitochondrial superoxides. As a consequence, these data suggest that mitochondria are crucial players in ferroptosis and that in our cells, the level of ferroptosis correlates with the degree of mitochondrial dysfunction.

### Lung adenocarcinoma CSCs show an increased expression of a set of antioxidant genes

**Fig. 3:**
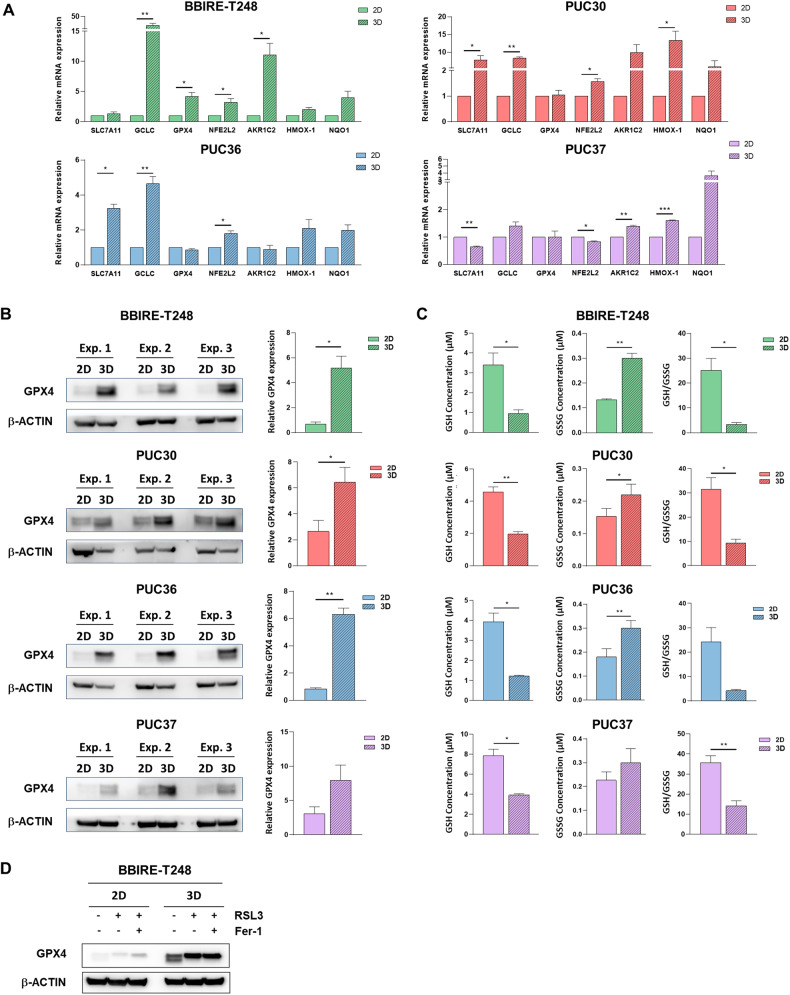
LUAD-CSCs show an increased expression of a set of antioxidant genes. **A** Gene expression of antioxidant genes by RT-PCR in BBIRE-T248, PUC30, PUC36, and PUC37 cell lines. Data from three independent experiments are expressed as fold change of the mean of gene expression ± SEM and are statistically significant if *p < 0.05 and very significant if **p < 0.01 (paired Student’s *t*-test). β-Actin was used for normalization. SLC7A11 solute carrier family 7 member 11, GCLC glutamate-cysteine ligase catalytic subunit, GPX4 glutathione peroxidase 4, NFE2L22 nuclear factor erythroid-derived 2-like 2, AKR1C2 aldo-keto reductase family 1 member C2, HMOX-1 heme oxygenase-1, NQO1 NAD(P)H quinone dehydrogenase 1. **B** Western blotting analysis of GPX4 and β-Actin in the four LUAD primary cell lines analysed. The panels show the bands of three independent experiments and the relative fold change of densitometric analyses of GPX4 and β-actin proteins. **C** Intracellular content of GSH and GSSG in BBIRE-T248, PUC30, PUC36, and PUC37 cell lines. Data from three independent experiments are expressed as mean ± SEM and are statistically significant if *p < 0.05 and very significant if **p < 0.01 (paired Student’s *t*-test). **D** Western blotting analysis of GPX4 and β-ACTIN in the LUAD primary cell line BBIRE-T248, following treatment with 1 μM RSL3 alone or in combination with Ferrostatin-1 (Fer-1) for 2 h.

Given the importance of antioxidant defense systems in the resistance of cancer stem cells to ferroptotic cell death, we investigated at the molecular level the mechanisms implemented by 3D cells to protect themselves from stress. Therefore, we carried out a molecular characterization of primary cell cultures, by evaluating the expression of antioxidant genes, namely Nfe2l2 (encoding for NRF2), Hmox-1, Nqo1, and Akr1c2, and genes related to glutathione metabolism such as Slc7a11, Gclc, and Gpx4. In Fig. 3A are reported the graphs of the relative mRNA expression as fold changes observed in the primary cell lines in the transition from 2D to 3D growth condition. We noticed that each cell line grown in 3D conditions showed a statistically significant overexpression of genes involved in antioxidant defense systems but with a different pattern of increased genes. Notably, this upregulation trend was particularly pronounced in BBIRE-T248 and PUC30 cell lines, which harbor only mutations in the Kras gene (Table 1) and show the greatest difference in terms of sensitivity to RSL3 between 2D and 3D (Fig. 1C). In contrast, in PUC36 e PUC37 cell lines the fold changes of antioxidant genes analysed were less marked. However, it is evident, in general, that 3D cultures trigger a mechanism to detoxify cells and to escape from cell death.

GPX4 is a core enzyme that regulates lipid peroxidation and therefore plays a critical role in maintaining lipid homeostasis. GPX4 is a selenoprotein with a dual role: converts its cofactor GSH (glutathione reduced form) to GSSG (glutathione disulfide oxidized form) and reduces phospholipid hydroperoxides (PLOOHs) to their corresponding alcohol (PLOHs) to avoid cell damage [25]. Since GPX4 is a key regulator of ferroptosis, we investigated, by western blotting, the expression levels of this enzyme in the four primary cell cultures analysed in this study. As shown in Fig. 3B, a marked overexpression of GPX4 is common to all primary cell lines, although mRNA levels did not show consistent modulations (Fig. 3A). We then investigated the intracellular content of GSH and GSSG through a luminescence assay and we observed that the levels of GSH decreased in all cell lines in the passage from 2D to 3D grown condition, while the GSSG content showed an opposite trend (Fig. 3C). We hypothesize that in 3D grown condition, the decreased levels of GSH are due to high expression of GPX4 that uses it as cofactor in enzymatic reaction, converting it into GSSG.

To understand the molecular mechanisms involved in the induction of ferroptosis in 2D vs 3D cells and to explore the role of GPX4 in this context, we treated the BBIRE-T248 cell line with 1 μM RSL3 alone or in combination with 2 μM Ferrostatin-1 for 2 h and evaluated the expression of GPX4. Results obtained by western blotting confirmed that GPX4 is strongly upregulated in 3D spheroids compared to the 2D cells, without substantial modulation following treatment. At a closer inspection, however, we observed that in the untreated cells, GPX4 is present with two isoforms: mitochondrial (the higher band) and cytosolic (the lower band). After treatment with RSL3, the cytosolic isoform seems to disappear, and we know that in 2D cells, this is sufficient to induce ferroptotic death (Fig. 3D). The same phenomenon is observed in the 3D spheroids, where, however, the mitochondrial isoform of GPX4 seems to increase following RSL3 treatment, to compensate for the absence of the cytoplasmic isoform. This suggests that 3D cells, in addition to having basally higher levels of GPX4 compared to their 2D counterpart, which confers lower sensitivity to RSL3, undergo also GPX4 isoform switching, which further enhances the anti-ferroptotic effect.

### Lung adenocarcinoma CSCs modulate other molecular pathways potentially involved in ferroptosis

**Fig. 4:**
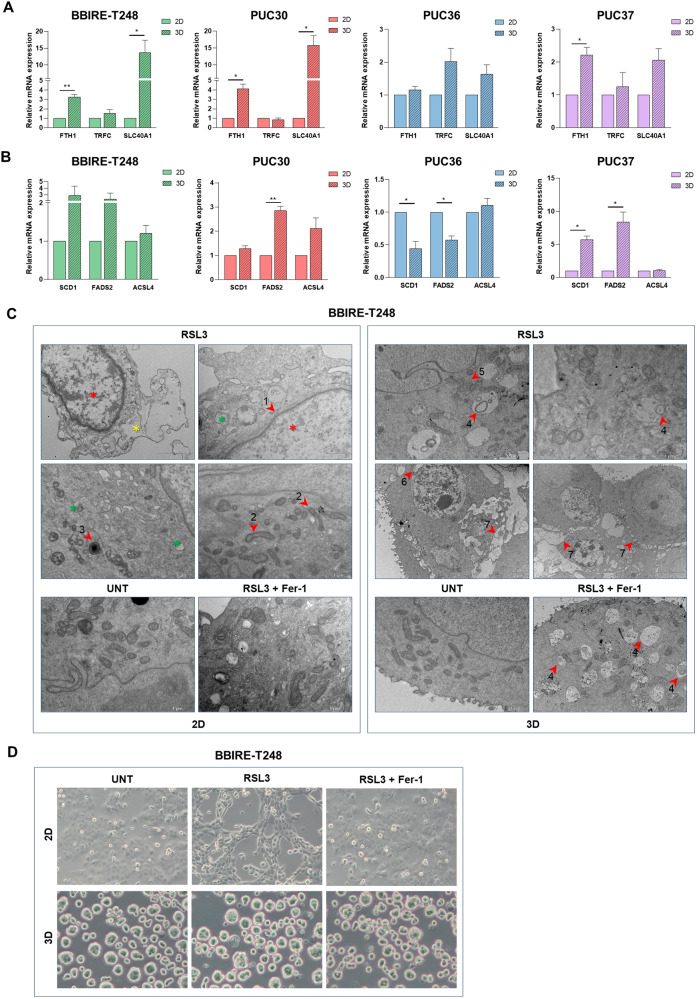
LUAD-CSCs modulate other molecular pathways potentially involved in ferroptosis. **A**, **B** Gene expression of genes involved in iron metabolism (**A**) and lipid metabolism (**B**) by RT-PCR in BBIRE-T248, PUC30, PUC36, and PUC37 cell lines. Data from three independent experiments are expressed as fold change of the mean of gene expression ± SEM and are statistically significant if *p < 0.05 and very significant if **p < 0.01 (paired Student’s *t*-test). β-actin was used for normalization. FTH1 Ferritin heavy chain 1, TRFC Transferrin receptor 1, SLC40A1 Solute Carrier Family 40 Member 1, SCD1 Stearoyl-CoA desaturase 1, FADS2 Fatty acid desaturase 2, ACSL4 Acyl-CoA synthetase long-chain family member 4. **C**, **D** Representative images of morphological aspect of BBIRE-T248 cells after treatment with RSL3 (1 μM) alone or in combination with Ferrostatin-1 (2 μM) for 2 h by transmission electron microscopy (**C**) and by inverted microscope (**D**). UNT untreated, Fer-1 Ferrostatin-1. (10x microscopic enlargement).

Another mechanism to escape ferroptotic cell death is to maintain iron homeostasis in the cell [26]. Storage, outflow, or inflow of iron makes cells more or less susceptible to ferroptosis [27]. For this reason, we assessed the different expression of iron metabolism-related genes in cells grown in 3D conditions versus 2D conditions by RT-PCR. In Fig. 4A is shown that the expression of Ferritin heavy chain 1 (FTH1) and Ferroportin-1 (also known as SLC40A1), responsible for iron storage and export, respectively, increased in 3D condition. Similarly to the antioxidant genes, this upregulation is more accentuated in BBIRE-T248 and PUC30, which show the greatest degree of sensitivity shift between 2D and 3D. Accordingly, with these data, our analysis suggests that in 3D conditions potentially toxic effects of iron are under control. Transferrin receptor 1 (TfR1) instead is responsible for Transferrin-bound iron (Tf-Fe3+) uptake concurring for cell iron accumulation and does not show significant differences in gene expression (TRFC).

It is known that lipid metabolism plays a crucial role in CSCs, providing the energy needed for their survival [23, 28] and affording protective mechanisms against peroxidation. The production of lipid droplets, for example, keeps Poly-Unsatured Fatty Acids (PUFAs) away from lipid oxidative damage [29], while lipid desaturation, regulated by the enzyme Stearoyl-CoA desaturase 1 (SCD1), decreases levels of PUFAs [16]. On the contrary, the enzyme Acyl-CoA synthetase long-chain family member 4 (ACSL4), that catalyzes the biosynthesis of polyunsaturated fatty acid-containing lipids, promotes the accumulation of lipid peroxidation products, leading to ferroptosis [30]. We then evaluated the expression of lipid metabolism genes in our 2D and 3D cellular models by quantitative RT-PCR. SCD1 and FADS2 (Fatty Acid Desaturase 2) resulted to be overexpressed in BBIRE-T248, PUC30, and PUC37 cell lines when cultured in 3D conditions, while ACSL4 expression was quite stable in all cell cultures comparing the two growth conditions (Fig. 4B). This suggests that lipid metabolism plays an important role in controlling CSCs maintenance, making cells less sensitive to peroxidation and cell death.

In order to identify other possible mechanisms involved in the protection against ferroptosis in 3D cells, we relied on microscopy analyses. In particular, we performed an ultrastructural investigation of organelles and subcellular compartments by means of a transmission electron microscope (TEM). The images in Fig. 4C represent BBIRE-T248 cells grown in 2D and 3D conditions, treated according to the previously described experimental scheme (Fig. 2A), and analysed by TEM. RSL3-treated 2D samples have clear characteristic marks of ferroptotic cell death. In particular, electron-bright nuclei (red asterisks) with damage to the outer membrane (red arrow number 1), dilated endoplasmic reticulum compatible with reticular stress (green asterisks), and mitochondria with flattened cristae are found (red arrow number 2). The phenomenon of ferroptosis in the 2D samples progressed to damage the cytoplasm as well (yellow asterisk). Numerous non-homogeneous lipid droplets are noted, indicating a possible heterogeneous content of these organelles (red arrow number 3). Cells grown as spheroids and treated with RSL3 show evident activation of autophagy for the presence of many autophagosomes (red arrow number 4). Probably, massive activation of autophagy could be a defense mechanism of the cells against ferroptotic cell death. Mitochondria are in contact with the endoplasmic reticulum cisternae indicating intense cross-talk among these organelles that can imply different processes such as lipid synthesis and/or lipid metabolism, modulation of mitochondrial morphology (fission and fusion), endoplasmic reticulum (ER) stress, autophagy, and Ca^2+^ handling (red arrow number 5). In some places, the inner and outer membranes of the nucleus are separated (red arrow number 6). Finally, we noticed that in the internal part of the spheroid, there is a partial loss of cell-cell junctions (red arrow number 7), which is partially restored in the samples treated with Ferrostatin-1. The sample treated with Ferrostatin-1 maintains a high number of autophagosomes compared to the control (red arrow number 4).

We were able to appreciate this different sensitivity to ferroptosis activator also by looking at morphological changes of the cells through inverted microscopy. As shown in Fig. 4D, BBIRE-T248 cells showed a loss of adherent and occlusive junctions between cells after treatment when grown in 2D conditions. This phenomenon was reverted when cells were cotreated with antioxidant Ferrostatin-1 because they showed a similar level of attachment as in control. Contrariwise, the same cells cultured in 3D conditions showed no morphological alterations compared to the control.

### Lung adenocarcinoma CSCs overexpress GGT1, SEPW1, and MUC1 genes that potentially confer ferroptosis protection

**Fig. 5:**
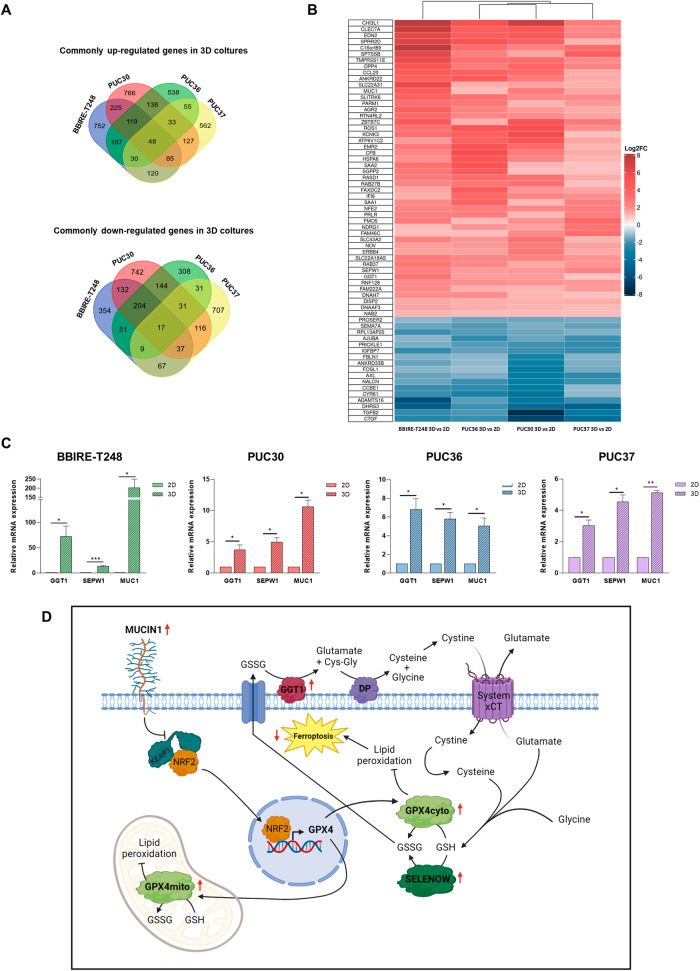
Differential expression analysis (DEA) of genes transcriptionally modulated in 3D spheroids versus 2D cells in BBIRE-T248, PUC36, PUC30, and PUC37 primary cell lines. **A** Venn diagram of commonly up and downregulated genes. **B** Heatmap of commonly up and downregulated genes. Only genes with log2Fold Change (FC) >1 or <−1 and adjusted *p*_val <0.05 were considered significantly upregulated (red) or downregulated (blue). **C** Gene expression of selected genes commonly upregulated in 3D spheroids of the four primary cell cultures (BBIRE-T248, PUC30, PUC36, and PUC37) by RT-PCR. Data from three independent experiments are expressed as fold change of the mean of gene expression ± SEM and are statistically significant if *p < 0.05 and very significant if **p < 0.01 (paired Student’s *t*-test). β-actin was used for normalization. GGT1 Gamma-glutamyltransferase 1, SEPW1 Selenoprotein W1, MUC1 Mucin 1. **D** Schematic model showing the putative role that each commonly upregulated gene in 3D cultures of the four tested primary lines may play in the protection from ferroptosis (Created with BioRender.com).

To finely characterize our established collection of patient-derived CSCs and identify common putative genes involved in driving the phenomenon of ferroptosis resistance, the four selected LUAD primary cell lines (BBIRE-T248, PUC30, PUC36, and PUC37) were profiled by RNA-sequencing, comparing the 2D and 3D in vitro culture systems (Tables S1–S4). In order to have an overview of the biological mechanisms mainly implicated in the 2D/3D transition, we performed a Gene Ontology analysis on the genes differentially expressed between the two growth conditions, employing the Auto-Go package [31]. Figure S3 shows, for each primary cell line, the GO biological processes enrichment analysis performed on upregulated genes (left panels) and downregulated genes (right panels) in 3D cells versus 2D cells. We found a set of enriched terms related to lipid metabolism, inflammation, and cytoskeleton organization for the upregulated genes, and to cell cycle and DNA replication for the downregulated ones. These results suggest that the transcriptional rewiring required by the 2D/3D transition involves core cellular processes, which do not allow finer biological mechanisms like ferroptosis to be among the top-order processes in enrichment analyses. We therefore adopted an investigative approach aimed at identifying individual genes, rather than entire processes, commonly modulated in the four cell lines. As shown in Fig. 5A, 48 genes were found to be commonly upregulated in the four cell cultures grown in 3D conditions compared to 2D counterpart, while the commonly downregulated genes were 17. Interestingly, among the common downregulated genes, Axl, Ajuba, Cyr61, and Ctgf are downstream genes of YAP/TAZ, two important transcription factors that positively regulate ferroptosis [32]. Among the commonly upregulated genes in 3D samples, we identified three genes which could contribute to ferroptosis protection, namely Sepw1, Muc1, and Ggt1. Sepw1 encodes for Selenoprotein W (SELENOW) that plays a role as a glutathione-dependent antioxidant and may be involved in redox-related processes [33]; Mucin 1 (MUC1), instead, might inhibit ferroptosis through Keap1-Nrf2-GPX4 pathway [34] and, finally, Gamma-Glutamyltransferase 1 (GGT1), an enzyme involved in the metabolism of glutathione (cofactor of GPX4), could confer resistance to ferroptosis regulating the activity of GPX4 (Fig. 5B) [35]. To validate this finding, we measured the expression levels of Ggt1, Sepw1, and Muc1 by RT-PCR analyses. In Fig. 5C are reported the graphs of the relative mRNA expression as fold changes observed in all primary cell lines in the transition from 2D to 3D growth condition. Data confirmed the upregulation of Ggt1, Sepw1, and Muc1 genes in 3D spheroids and suggested that, since GGT1 provides substrates for glutathione synthesis, which is utilized as cofactor of both GPX4 and Selenoprotein W, lung CSCs may be protected from ferroptosis by the activation of a GSH-dependent antioxidant mechanism. Based on the commonly upregulated genes in 3D spheroids and their known function in the literature, we hypothesized a molecular model consisting of a limited number of proteins, in which each of them may contribute partially to confer resistance to ferroptotic cell death (Fig. 5D).

## Discussion

CSCs have gained increasing attention in cancer research over the last two decades because they have been associated with resistance to conventional therapies and the development of tumor recurrences [36]. In recent years it has been recognized that the presence of cells with CSCs properties is linked to the phenomenon of cell plasticity by which epigenetic changes occurring in certain cancer cells can lead them to switch their phenotype between a less differentiated status into a more differentiated one and vice versa [7]. Ferroptosis, a non-apoptotic cell death process driven by aberrant metabolism and iron-dependent lipid peroxidation, has been implicated in a variety of pathologies, including cancer [3, 37]. It has been suggested that the induction of ferroptosis may represent a new therapeutic strategy to target CSCs in different types of cancer [6]. These findings prompted us to investigate more in depth this phenomenon in lung adenocarcinoma, to identify novel potential markers responsible for resistance to ferroptotic death in CSCs. To study lung adenocarcinoma, we used an experimental model already widely adopted in our laboratory, based on the establishment of primary cell cultures of tumor cells from MPEs of patients with metastatic LUAD, then cultured as 3D spheroids in order to enrich for CSCs [8, 9].

The most important finding of the study was the observation that lung adenocarcinoma primary cell cultures grown in 3D conditions underwent a phenotypic switch in terms of sensitivity to the ferroptosis-inducing agent RSL3, when compared to cells grown in 2D conditions. More precisely, the four primary cultures grown in 2D conditions were all sensitive to RSL3, although with a variable sensitivity, which nicely correlated with their mutational status. Indeed, in accordance with the literature, primary cells bearing mutations in Keap1 and/or Tp53 genes were more resistant to RSL3 as compared to cultures without these mutations [14, 22]. In contrast, when grown as 3D spheroids in a serum‐free medium supplemented with growth factors, which favors the enrichment of CSCs markers, all primary cells, independently from their genetic makeup, acquired strong resistance to ferroptotic cell death. Interestingly, disrupting 3D cells and growing them in adherence reversed this occurrence of resistance, confirming the notion of CSCs plasticity, which maintains that cancer cells have the dynamic potential to switch between a CSC state and a non-CSC state.

The mechanism and functional roles of this phenomenon of phenotypic plasticity are related to epigenetic changes having effects on gene expression patterns and key pathways connected with cell proliferation and survival. In particular, 3D spheroids underwent a series of molecular changes that probably help to detoxify cells by increasing expression of a set of antioxidant genes, first of all, GPX4, an enzyme functional to repair oxidative damage to lipids and therefore leading inhibitor of ferroptosis [25]. It is known that GPX4 selenoprotein level is regulated transcriptionally by transcription factor NRF2 and its activity can be upregulated by supplementing intracellular selenium or glutathione, while it can be inhibited by ferroptosis inducers such as RSL3 [38]. The fine mechanisms that regulate Gpx4 show great potential for treating ferroptosis-related cancer [39]. In this context, the marked upregulation of GPX4 observed in 3D spheroids, accompanied by a significant consumption of its cofactor GSH, strongly correlated with the lack of formation of lipid peroxides and mitochondrial superoxides in RSL3-treated cells. Furthermore, the observation that the mitochondrial GPX4 isoform underwent an increased expression after treatment with RSL3 would suggest the activation of a compensatory mechanism based on GPX4 “isoform switching” capable of supporting and amplifying the anti-ferroptotic activity. Since ferroptosis is an important process of cell death in various conditions, and GPX4 is its core regulator, further studies will be needed to understand how GPX4 is regulated in 3D spheroids, i.e., whether transcriptionally, post-transcriptionally, post-translationally or a combination of these regulatory mechanisms.

Transcriptomic analyses resulting from the bulk RNA-sequencing of the primary cultures also allowed us to identify differentially expressed genes in the 3D vs 2D comparison, with the aim of establishing the molecular mechanisms that orchestrate the phenomenon of ferroptosis resistance in LUAD-CSCs. In particular, we focused attention on the 48 commonly upregulated genes in cells grown in 3D conditions compared to their 2D counterpart, and based on their known function in the literature, we selected three genes, Ggt1, Sepw1, and Muc1, which together to GPX4 may contribute to give rise to an aggressive and drug-resistant phenotype (Fig. 5D). Selenoprotein W, in fact, encoded by the Sepw1 gene, is a selenium-containing protein with glutathione-dependent antioxidant activity, and in this biological context it could enhance the detoxifying effect operated by GPX4. The role of GGT1 is to actively contribute to the GSH synthesis process, which could be essential for the enzymatic functions of both GPX4 and Selenoprotein W. This hypothesis is strengthened by the evidence that the disruption of GGT1 is sufficient to induce ferroptosis [35]. Mucin 1, instead, is a transmembrane glycoprotein overexpressed in non-small cell lung cancer [40]. It has been recently demonstrated that the inhibition of Muc1 hinders the entry of NRF2 into the nucleus, reducing the expression level of GPX4 and triggering ferroptosis [34]. In line with this observation, the high expression of Muc1 in 3D spheroids suggests a role in inhibiting ferroptosis through the Nrf2-GPX4 pathway.

Overall, our data suggest that lung CSCs-enriched 3D spheroids strengthen their antioxidant response compared to the 2D counterpart, resulting therefore protected from ferroptosis by the activation of a GSH-dependent detoxification mechanism. 3D spheroids constitute thus a good model for studying resistance to ferroptosis in lung adenocarcinoma, and the subset of genes discovered in this study would require deeper investigation to define new therapeutic approaches to defeat this type of cancer.

In conclusion, we believe that the present study can have a significant impact on our understanding of the mechanisms of resistance to therapies in lung adenocarcinoma. The results obtained could in fact open new avenues not only to develop new pharmacological therapies with consequent long-term disease control term, but also to identify new and promising biomarkers capable of predicting the onset of drug resistance.

## Materials and methods

### Sample collection, processing, and cultures

Non-small cell lung cancer (NSCLC) established human cell lines NCI-H460, NCI-H358, NCI-H1373, and NCI-H1792 were obtained from American Type Culture Collection (ATCC, Manassas, VA, USA) and were cultured according to the manufacturer’s instructions. BBIRE-T248 primary culture was isolated from the MPEs of a patient with LUAD at the Regina Elena National Cancer Institute (IRE) [9]. Cell lines named PUC, followed by a progressive number, were isolated from MPEs of patients with LUAD at Policlinico Umberto I, Sapienza University of Rome. The study was approved by the IFO Institutional Research Ethics Committee (Prot. Number 1032/17) and Sapienza University Ethics Committee (Prot. Number 210 SA_2020; Prot. Number 0946/2021); all patients agreed to participate in the study by signing an informed consent form. MPEs were obtained by thoracentesis and collected aseptically in collection bags or drainage containers. The tumor cell isolation protocol involves several steps, already previously described [41]. Cells were cultured in adherence in RPMI‐1640 medium supplemented with 10% FBS, 1% l-glutamine, 1% Penicillin-Streptomycin (Sigma) at 37 °C and 5% CO_2_. The 3D spheroids were cultured in ultra‐low attachment flasks (Corning, NY) in “sphere medium” composed by serum‐free DMEM/F12 supplemented with fibroblast growth factor-basic (b-FGF, 20 ng/ml), epidermal growth factor (EGF, 20 ng/ml), insulin (20 μg/ml), d-glucose (0,5%), heparin (5 μg/ml), l-glutamine (2,5 mM), Penicillin-Streptomycin (0,1%) (Sigma), and B27 (0,1%) (Gibco, Invitrogen, Carlsbad, CA). All cells were tested with Mycoplasma PCR Detection Kit (abm, Cat.No. G238) and subjected to short tandem repeat (STR) analyses by ATCC cell line authentication service. The images of the cells were acquired with Axiocam 208 color (Zeiss), a digital camera coupled with a Primovert microscope (Carl Zeiss).

### Characterization of primary lines by flow cytometry

To detect surface markers, 300,000 cells were incubated for 30 min at 4 °C with anti-CD45, anti-CD90, and anti-EpCAM antibodies. To detect the intracellular marker CK7/8, instead, 500,000 cells were fixed with 1% Formalin solution (Sigma) in PBS and permeabilized with 0.2% Triton X-100 (Sigma) in PBS; afterwards, cells were incubated for 30 min at 4 °C with Anti-CK7/8 anti-antibody. The fluorescent signal was detected following the indications of antibody manufacturers with CytoFLEX flow cytometer (Beckman Coulter).

### Mutation analysis by next-generation sequencing (NGS)

The Ion Torrent^TM^ Oncomine^TM^ Focus Assay was used for next-generation sequencing (NGS). This assay enables the detection of variants in 52 genes relevant to solid tumors allowing concurrent analysis of DNA and RNA to simultaneously detect multiple types of variants, including hotspots, single-nucleotide variants (SNVs), indels, copy number variants (CNVs), and gene fusions, in a single workflow.

### Quantitative real-time PCR analysis

Total RNA was isolated by TRIzol Reagent (Life Technologies, Gaithersburg, MD) following the manufacturer’s instructions [42]. RNA was reverse transcribed into cDNA using PrimeScript RT Reagent Kit with gDNA Eraser (Takara Bio Inc, Kusatsu, Shiga, Japan). The qRT-PCR was performed using SYBR green detection (Applied Biosystem; Life Technologies, Gaithersburg, MD) in a 7500 StepOnePlus (Applied Biosystems, Foster City, CA, USA). The comparative method (2^–ΔΔCt^) was used for relative quantification, and β-actin was used for normalization. Three independent experimental replicates were performed and graphed as fold change of the mean ± standard error of the mean (SEM) with GraphPad Prism 8 software.

### Western blot analysis

Cells were lysed in RIPA Buffer (Sigma) containing the Phosphatase Inhibitor Cocktail and Protease Inhibitor Cocktail (Roche), and kept on ice for 40 min vortexing every 5 min. Afterward, samples were centrifuged at 13,000 rpm for 25 min at 4 °C. Cell lysates were quantized with Protein Assay Dye Reagent Concentrate (Bio-Rad) and separated by SDS-PAGE with Bolt Bis-Tris Plus gels (4–12% polyacrylamide concentrations) in MES SDS Running Buffer (Invitrogen). PageRuler™ Prestained Protein Ladder (Thermo Scientific™) was used as size standards of proteins. The proteins were transferred on nitrocellulose membrane using the iBlot 2 Dry Blotting System with iBlot 2 Transfer Stacks (Invitrogen). Membranes were blocked for 1 h with 5% Milk (Skim Milk Powder, Millipore) in TBS-Tween 0,1% (TBS, Bio-Rad; Tween 20, Sigma-Aldrich) and then incubated overnight with anti-GPX4 (Cell Signaling, #52455) or anti-β-actin (Sigma, A5441) primary antibodies. The next day, membranes were incubated with secondary antibody HRP-conjugated and developed with ECL West Pico Plus Substrate (Immunological Sciences). Images were acquired with Azure c300 Gel Imaging System (Azure Biosystems). All results were normalized over β-actin.

### Drug treatments and viability assays

Cells were plated in 96-well plates: 6000 cells/well for primary cells, 3000 cells/well for NCI-H460 cell line, 8000 cells/well for NCI-H358 and NCI-H1792 cell lines, 10,000 cells/well for NCI-H1373 cell line. The 3D cells were cultured in 96-well plates with ultra‐low attachment surfaces (Corning, NY) in a sphere medium. For all cell lines, both 2D and 3D, viability was assessed using CellTiter-Glo 3D (Promega), and the luminescence was measured to GloMax Explorer luminometer (Promega). After 72 h of treatment with 1 S,3R-RSL3 (Sigma), the half maximal inhibitory concentration (IC_50_) value was measured, testing drugs at 1:3 scalar concentrations (0.008–50 μM) and calculated with GraphPad Prism 8 software. The dose-response curves refer to three independent experiments and were represented as mean ± SEM.

### Flow cytometry analyses

Cells were seeded in a 6-well plate (250,000 cells per well) and treated for 2 h with RSL3 alone or in combination with Ferrostatin-1 (Sigma). The untreated condition is to be considered treated with the maximum dose of DMSO used in the experiment. After treatments, lipid peroxidation was investigated, incubating cells for 30 min at 37 °C with BODIPY™ 581/591 C11 (Thermo Fisher Scientific) diluted 1:1 000. Mitochondrial ROS were quantified incubating cells for 20 min at 37 °C with MitoSOX Red Mitochondrial Superoxide Indicator (Thermo Fisher Scientific) diluted 1:1000. The 3D cells were dissociated with Accumax solution (Sigma) before incubation with reagents. Fluorescence was analysed by flow cytometry, according to the instructions of the manufacturer. Fluorescence was detected with a CytoFLEX flow cytometer (Beckman Coulter), and a minimum of 10,000 cells were analysed. The dead cells of each sample were identified with SYTOX™ Blue Dead Cell Stain (Invitrogen) diluted 1:2000. Each experiment was performed in triplicate and data were analysed with FlowJo software.

### Confocal analysis

BBIRE-T248 samples stained with Bodipy™ 581/591 C11 (see previous paragraph) were fixed with 4% paraformaldehyde (Thermo Fisher Scientific), cytocentrifuged to slides, and observed using a Zeiss Confocal Microscope (ZEISS LM900, Germany). Optical spatial series with a step size of 1 micron have been acquired, and the orthogonal projection image of each series has been generated using the Zen 3.0 Blue Edition software.

### RNA-sequencing

Total RNA was extracted using Qiazol (Qiagen, Hilden, Germany), purified from DNA contamination through a DNase I (Qiagen) digestion step, and further enriched by Qiagen miRNeasy columns profiling (Qiagen). Quantity and integrity of the extracted RNA were assessed by Nanodrop Spectrophotometer (Nanodrop Technologies LCC, Thermo Fisher, Waltham, MA, USA) and by Agilent 2100 Bioanalyzer (Agilent Technologies, Santa Clara, CA, USA), respectively. RNA libraries for sequencing were generated using the TruSeq RNA Exome kit (Illumina, San Diego, CA, USA), which is perfectly suited for low input and low-quality RNA. The procedure consists of two steps, the first one is a general whole transcriptome strand-specific library preparation followed by a specific exon targeting enrichment. The quality of the resulting libraries was assessed via Bioanalyzer (High Sensitivity DNA Kit). The intermediate library, before exon enrichment, was quantified by Qubit, and the final library by qPCR. Samples were sequenced in paired-end mode, sequencing 76 bp from each side, with NextSeq 500 System (Illumina).

RNA-seq raw data were processed thanks to the nf-core/rnaseq pipeline (v 2.4), which carries out the primary analyses of the mapping onto the reference genome (GRCh37), providing quality control metrics of the analysed samples. Both the raw counts (FeatureCounts) and the TPM (transcript per million) normalized pseudo counts from the Salmon tool were obtained from the pipeline. The DEseq2 Bioconductor package was used to test for the differentially expressed genes (DEGs) between the groups using the negative binomial distribution and Wald’s test. For DEG identification, the cut-off criteria of |log2FC| >1 and adjusted *p* value <0.05 were considered statistically significant (Tables S1–S4). The package AUTO-go was employed to automatize Differential Expression analyses across all conditions [31]. The biological function of DEGs was identified by Gene Ontology (GO) analysis using the R package “enrichR”. GO visualizations were produced via the auto-GO/ARGO framework. Fisher’s exact test was employed, and the occurrence of false positives was corrected by the Benjamini–Hochberg (B-H) multiple-test correction method. An adjusted *p* value <0.05 was set as the cut-off criterion.

### GSH/GSSG-Glo™ assay

The GSH/GSSG-Glo™ assay, a luminescence-based system, was used to detect and quantify total glutathione (GSH + GSSG), GSSG and GSH-to-GSSG ratios in cultured cells. The assays are performed according to the manufacturer’s instructions and the luminescence was measured to GloMax Explorer luminometer (Promega).

### Transmission electron microscopy (TEM)

To evaluate cancer cell fine subcellular ultrastructural features, samples were fixed in glutaraldehyde 2.5%/paraformaldehyde 4% and prepared for transmission electron microscopy (TEM) purposes. Briefly, fixed samples were rinsed with cacodylate buffer for at least 1 h, post-fixed with 1% OsO4 in a cacodylate buffer, treated with tannic acid, dehydrated in ethanol, and embedded in epoxy resin. Ultrathin sections (60 nm) were treated with uranyl-acetate and then contrasted with lead hydroxide. Samples were studied with a transmission electron microscope Philips EM208S (FEI) with a tungsten filament as electron source and x200K maximum magnification; the image acquisition system is MegaView III (Olympus Soft Imaging Solutions).

### Statistical analysis

In vitro experiments were presented as mean ± standard error of the mean (SEM) of three independent experiments. Statistical differences between experimental groups were analysed by paired Student’s *t*-test, using GraphPad Prism 8.0.2 software. A *p* value (*p*) <0.05 was considered as statistically significant (*) and *p* < 0.01 as very significant (**).

### Supplementary information

Supplementary figures

Table S1

Table S2

Table S3

Table S4

Table S5

Original western blots

## Data Availability

All data generated or analysed during this study are included in this published article and its supplementary information files. Raw data are available upon reasonable request.

## References

[1] Leiter A, Veluswamy RR, Wisnivesky JP (2023). The global burden of lung cancer: current status and future trends. Nat Rev Clin Oncol.

[2] Kim D, Choi BH, Ryoo IG, Kwak MK (2018). High NRF2 level mediates cancer stem cell-like properties of aldehyde dehydrogenase (ALDH)-high ovarian cancer cells: inhibitory role of all-trans retinoic acid in ALDH/NRF2 signaling. Cell Death Dis.

[3] Zhang C, Liu X, Jin S, Chen Y, Guo R (2022). Ferroptosis in cancer therapy: a novel approach to reversing drug resistance. Mol Cancer.

[4] Dixon SJ, Lemberg KM, Lamprecht MR, Skouta R, Zaitsev EM, Gleason CE (2012). Ferroptosis: an iron-dependent form of nonapoptotic cell death. Cell.

[5] Dixon SJ, Stockwell BR (2019). The hallmarks of ferroptosis. Annu Rev Cancer Biol.

[6] Cosialls E, El Hage R, Dos Santos L, Gong C, Mehrpour M, Hamaï A (2021). Ferroptosis: cancer stem cells rely on iron until “to die for” it. Cells.

[7] Warrier NM, Kelkar N, Johnson CT, Govindarajan T, Prabhu V, Kumar P (2023). Understanding cancer stem cells and plasticity: towards better therapeutics. Eur J Cell Biol.

[8] Mancini R, Giarnieri E, de Vitis C, Malanga D, Roscilli G, Noto A, et al. Spheres derived from lung adenocarcinoma pleural effusions: Molecular characterization and tumor engraftment. PLoS ONE. 2011;6:e21320. 10.1371/journal.pone.0021320 PMC3138755 21789168

[9] Bruschini S, di Martino S, Pisanu ME, Fattore L, De Vitis C, Laquintana V (2020). CytoMatrix for a reliable and simple characterization of lung cancer stem cells from malignant pleural effusions. J Cell Physiol.

[10] De Vitis C, Battaglia AM, Pallocca M, Santamaria G, Mimmi MC, Sacco A (2023). ALDOC- and ENO2- driven glucose metabolism sustains 3D tumor spheroids growth regardless of nutrient environmental conditions: a multi-omics analysis. J Exp Clin Cancer Res.

[11] Noto A, Raffa S, De Vitis C, Roscilli G, Malpicci D, Coluccia P (2013). Stearoyl-CoA desaturase-1 is a key factor for lung cancer-initiating cells. Cell Death Dis.

[12] Pisanu ME, Noto A, De Vitis C, Morrone S, Scognamiglio G, Botti G (2017). Blockade of stearoyl-CoA-desaturase 1 activity reverts resistance to cisplatin in lung cancer stem cells. Cancer Lett.

[13] Tesfay L, Paul BT, Konstorum A, Deng Z, Cox AO, Lee J (2019). Stearoyl-CoA desaturase 1 protects ovarian cancer cells from ferroptotic cell death. Cancer Res.

[14] Wohlhieter CA, Richards AL, Uddin F, Hulton CH, Quintanal-Villalonga À, Martin A, et al. Concurrent mutations in STK11 and KEAP1 promote ferroptosis protection and SCD1 dependence in lung cancer. Cell Rep. 2020;33:108444. 10.1016/j.celrep.2020.108444 PMC7722473 33264619

[15] Luis G, Godfroid A, Nishiumi S, Cimino J, Blacher S, Maquoi E, et al. Tumor resistance to ferroptosis driven by stearoyl-CoA desaturase-1 (SCD1) in cancer cells and fatty acid biding protein-4 (FABP4) in tumor microenvironment promote tumor recurrence. Redox Biol. 2021;43:102006. 10.1016/j.redox.2021.102006 PMC8163990 34030117

[16] Ascenzi F, De Vitis C, Maugeri-Saccà M, Napoli C, Ciliberto G, Mancini R (2021). SCD1, autophagy and cancer: implications for therapy. J Exp Clin Cancer Res.

[17] Moin AT, Sarkar B, Ullah MA, Araf Y, Ahmed N, Rudra B (2021). In silico assessment of EpCAM transcriptional expression and determination of the prognostic biomarker for human lung adenocarcinoma (LUAD) and lung squamous cell carcinoma (LUSC). Biochem Biophys Rep..

[18] Kummar S, Fogarasi M, Canova A, Mota A, Ciesielski T (2002). Cytokeratin 7 and 20 staining for the diagnosis of lung and colorectal adenocarcinoma. Br J Cancer.

[19] Zeng F, Gao M, Liao S, Zhou Z, Luo G, Zhou Y (2023). Role and mechanism of CD90+ fibroblasts in inflammatory diseases and malignant tumors. Mol Med.

[20] Kim N, Kim HK, Lee K, Hong Y, Cho JH, Choi JW, et al. Single-cell RNA sequencing demonstrates the molecular and cellular reprogramming of metastatic lung adenocarcinoma. Nat Commun. 2020;11:2285. 10.1038/s41467-020-16164-1 PMC7210975 32385277

[21] Rheinländer A, Schraven B, Bommhardt U (2018). CD45 in human physiology and clinical medicine. Immunol Lett.

[22] Ji H, Wang W, Li X, Han X, Zhang X, Wang J (2022). p53: a double-edged sword in tumor ferroptosis. Pharm Res.

[23] Mancini R, Noto A, Pisanu ME, De Vitis C, Maugeri-Saccà M, Ciliberto G (2018). Metabolic features of cancer stem cells: the emerging role of lipid metabolism. Oncogene.

[24] Liu Y, Lu S, Wu Llei, Yang L, Yang L, Wang J (2023). The diversified role of mitochondria in ferroptosis in cancer. Cell Death Dis.

[25] Seibt TM, Proneth B, Conrad M (2019). Role of GPX4 in ferroptosis and its pharmacological implication. Free Radic Biol Med.

[26] Galy B, Conrad M, Muckenthaler M. Mechanisms controlling cellular and systemic iron homeostasis. Nat Rev Mol Cell Biol. 2023;25:133–55 10.1038/s41580-023-00648-1 37783783

[27] Venkataramani V (2021). Iron homeostasis and metabolism: two sides of a coin. Adv Exp Med Biol.

[28] Visweswaran M, Arfuso F, Warrier S, Dharmarajan A (2020). Aberrant lipid metabolism as an emerging therapeutic strategy to target cancer stem cells. Stem Cells.

[29] Tirinato L, Pagliari F, Limongi T, Marini M, Falqui A, Seco J, et al. An overview of lipid droplets in cancer and cancer stem cells. Stem Cells Int. 2017;2017:1656053. 10.1155/2017/1656053 PMC5572636 28883835

[30] Doll S, Proneth B, Tyurina YY, Panzilius E, Kobayashi S, Ingold I (2017). ACSL4 dictates ferroptosis sensitivity by shaping cellular lipid composition. Nat Chem Biol.

[31] Sperandio E, Grassucci I, D’ambrosio L, Pallocca M. Automated, reproducible investigation of gene set Dif-1 ferential enrichment via the AUTO-go framework 2. bioRxiv [Preprint].

[32] Magesh S, Cai D (2022). Roles of YAP/TAZ in ferroptosis. Trends Cell Biol.

[33] Jeong Dwon, Kim TS, Chung YW, Lee BJ, Kim IY (2002). Selenoprotein W is a glutathione-dependent antioxidant in vivo. FEBS Lett.

[34] Wang YM, Gong FC, Qi X, Zheng YJ, Zheng XT, Chen Y, et al. Mucin 1 inhibits ferroptosis and sensitizes vitamin E to alleviate sepsis-induced acute lung injury through GSK3 β/Keap1-Nrf2-GPX4 pathway. Oxid Med Cell Longev. 2022;2022:2405943. 10.1155/2022/2405943 PMC9334047 35910848

[35] Hayashima K, Katoh H (2022). Expression of gamma-glutamyltransferase 1 in glioblastoma cells confers resistance to cystine deprivation–induced ferroptosis. J Biol Chem.

[36] Mayani H, Chávez-González A, Vázquez-Santillan K, Contreras J, Guzman ML (2022). Cancer stem cells: biology and therapeutic implications. Arch Med Res.

[37] Xiong R, He R, Liu B, Jiang W, Wang B, Li N, et al. Ferroptosis: a new promising target for lung cancer therapy. Oxid Med Cell Longev. 2021;2021:8457521. 10.1155/2021/8457521 PMC8487823 34616505

[38] Takahashi N, Cho P, Selfors LM, Kuiken HJ, Kaul R, Fujiwara T (2020). 3D culture models with CRISPR screens reveal hyperactive NRF2 as a prerequisite for spheroid formation via regulation of proliferation and ferroptosis. Mol Cell.

[39] Cui C, Yang F, Li Q. Post-translational modification of GPX4 is a promising target for treating ferroptosis-related diseases. Front Mol Biosci. 2022;9:901565. 10.3389/fmolb.2022.901565 PMC9133406 35647032

[40] Chen W, Zhang Z, Zhang S, Zhu P, Ko JKS, Kin-Lam Yung K. Molecular sciences MUC1: structure, function, and clinic application in epithelial cancers. Int J Mol Sci. 2021;22:6567. 10.3390/ijms22126567 PMC8234110 34207342

[41] Bruschini S, Pallocca M, Sperandio E, D’Ambrosio L, Ascenzi F, De Vitis C (2022). Deconvolution of malignant pleural effusions immune landscape unravels a novel macrophage signature associated with worse clinical outcome in lung adenocarcinoma patients. J Immunother Cancer.

[42] De Vitis C, D’ascanio M, Sacconi A, Pizzirusso D, Salvati V, Mancini M, et al. B4GALT1 as a new biomarker of idiopathic pulmonary fibrosis. Int J Mol Sci. 2022;23:15040. 10.3390/ijms232315040 PMC9738382 36499368

