# Supplementary material for: Identification of a set of genes potentially responsible for resistance to ferroptosis in lung adenocarcinoma cancer stem cells
Source: Cell Death Dis. 2024 Apr 29;15(4):303. doi: 10.1038/s41419-024-06667-w (PMC11059184; doi:10.1038/s41419-024-06667-w)
Supplement: Supplementary file 1 — Supplementary figures [file 41419_2024_6667_MOESM1_ESM.pdf]

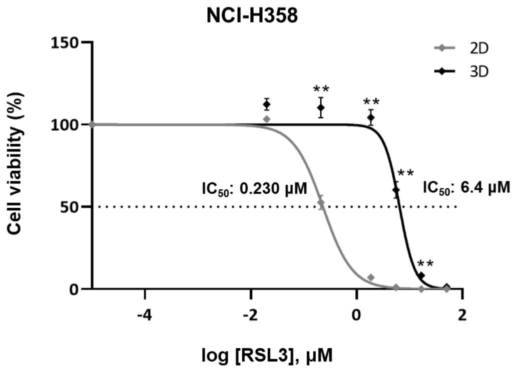
NCI-H358
2D
3D
Cell viability (%)
150
100
50
0
**
**
**
**
IC50: 0.230 μM
IC50: 6.4 μM
-4
-2
0
2
log [RSL3], μM

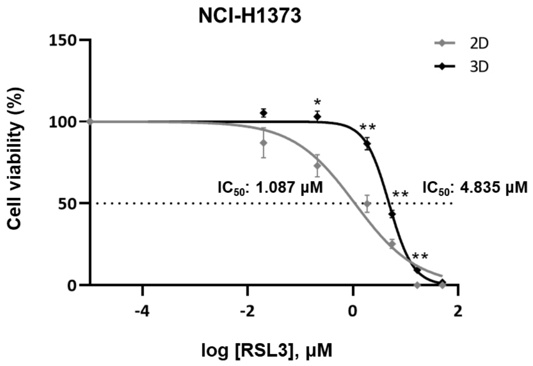
NCI-H1373
2D
3D
Cell viability (%)
150
100
50
0
*
**
**
**
IC50: 1.087 μM
IC50: 4.835 μM
-4
-2
0
2
log [RSL3], μM

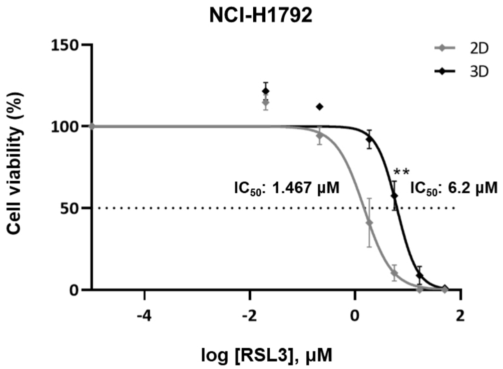
NCI-H1792
2D
3D
Cell viability (%)
150
100
50
0
**
IC50: 1.467 μM
IC50: 6.2 μM
-4
-2
0
2
log [RSL3], μM

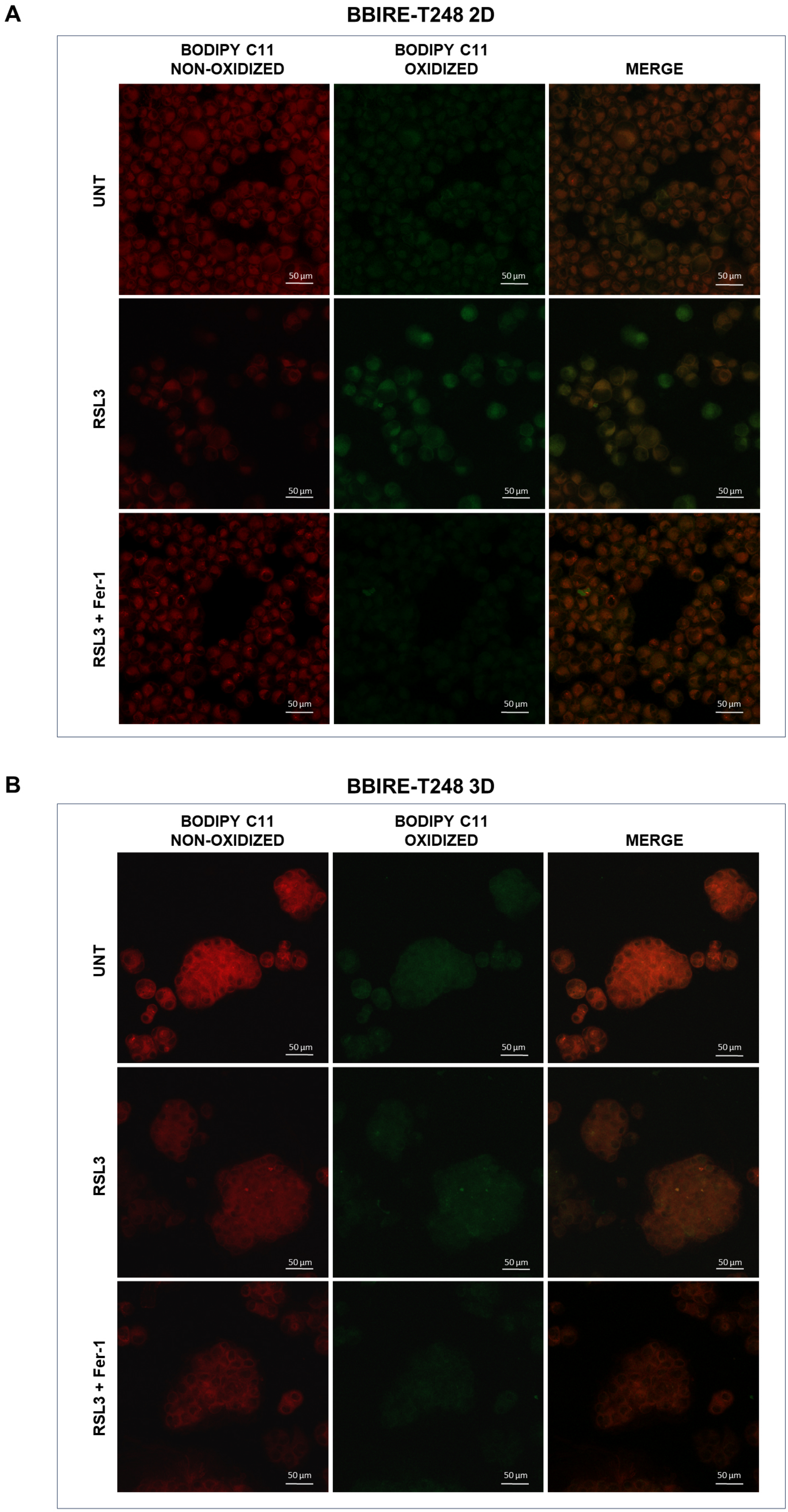
A
BBIRE-T248 2D
BODIPY C11 NON-OXIDIZED
BODIPY C11 OXIDIZED
MERGE
UNT
RSL3
RSL3 + Fer-1
50 µm
B
BBIRE-T248 3D
BODIPY C11 NON-OXIDIZED
BODIPY C11 OXIDIZED
MERGE
UNT
RSL3
RSL3 + Fer-1
50 µm

BBIRE-T248

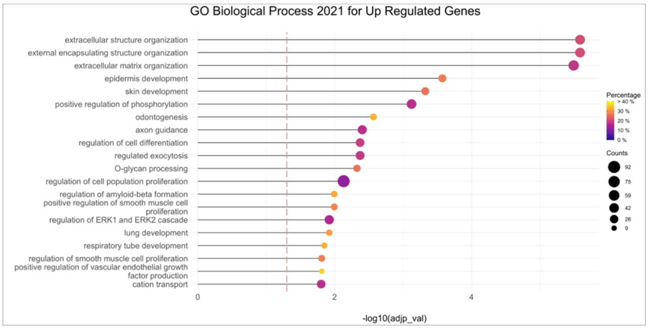

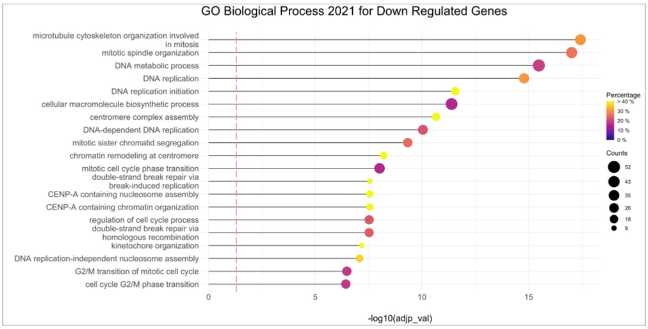

PUC30

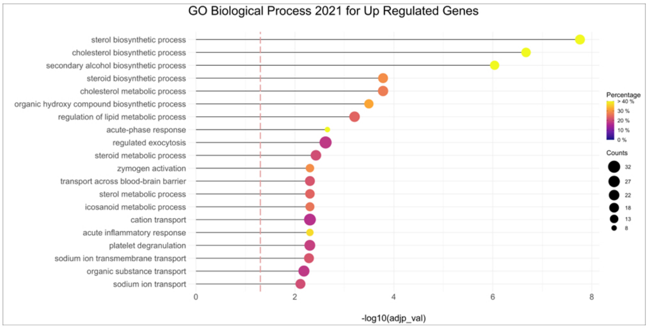

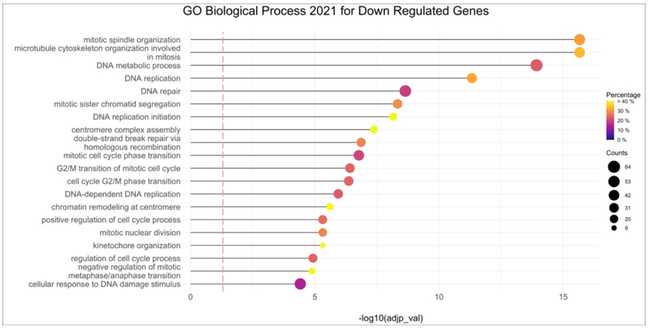

PUC36

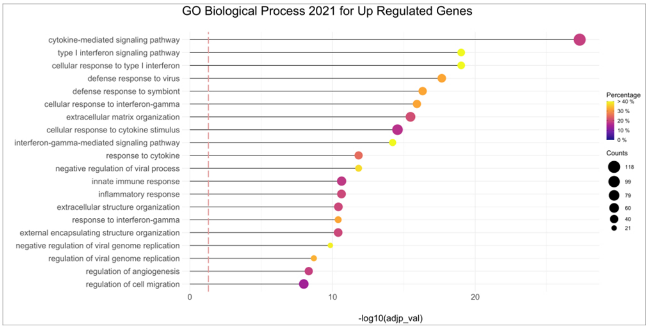

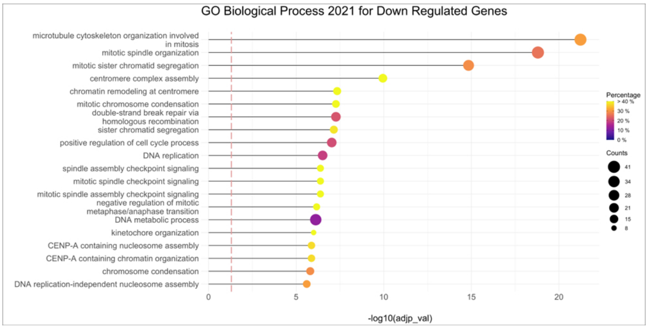

PUC37

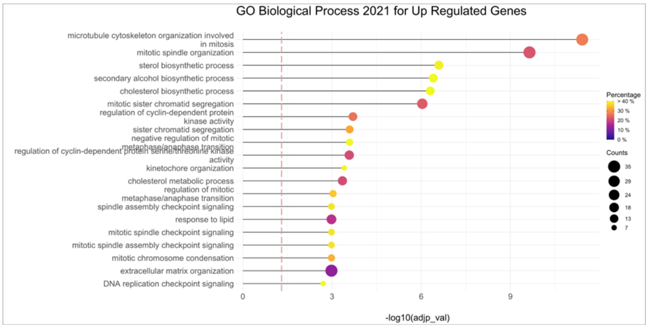

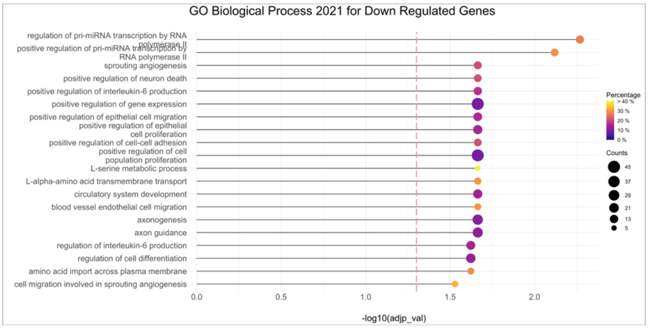

SUPPLEMENTARY FIGURE LEGENDS

Figure S1: Dose-response curves of sensitivity to RSL3 at 72h in NCI-H358, NCI-1373, and NCI-H1792 stable cell lines grown in 2D and 3D conditions. Data are representative of three independent experiments; values are expressed as mean ± SEM and are statistically significant if $*p < 0.05$ (Welch's t-test) for the comparison between 2D and 3D. The IC_{50} (half maximal inhibitory concentration) values are reported in the graphs.

Figure S2: Confocal imaging of BBIRE-T248 cells stained with Bodipy™ 581/591 C11 grown in 2D (A) or 3D (B) conditions and treated with RSL3 alone or in combination with Ferrostatin-1; Red, reduced form of C11-BODIPY; Green, oxidised form of C11-BODIPY.

Figure S3: Gene ontology (GO) analysis of upregulated genes (left panel) and downregulated genes (right panel) in 3D versus 2D cells. Terms over red dashed line are considered statistically significant (adjusted p-value<0.05).
