# Supplementary material for: Identification of a set of genes potentially responsible for resistance to ferroptosis in lung adenocarcinoma cancer stem cells
Source: Cell Death Dis. 2024 Apr 29;15(4):303. doi: 10.1038/s41419-024-06667-w (PMC11059184; doi:10.1038/s41419-024-06667-w)

Relative to Figure 3 B

BBIRE-T248

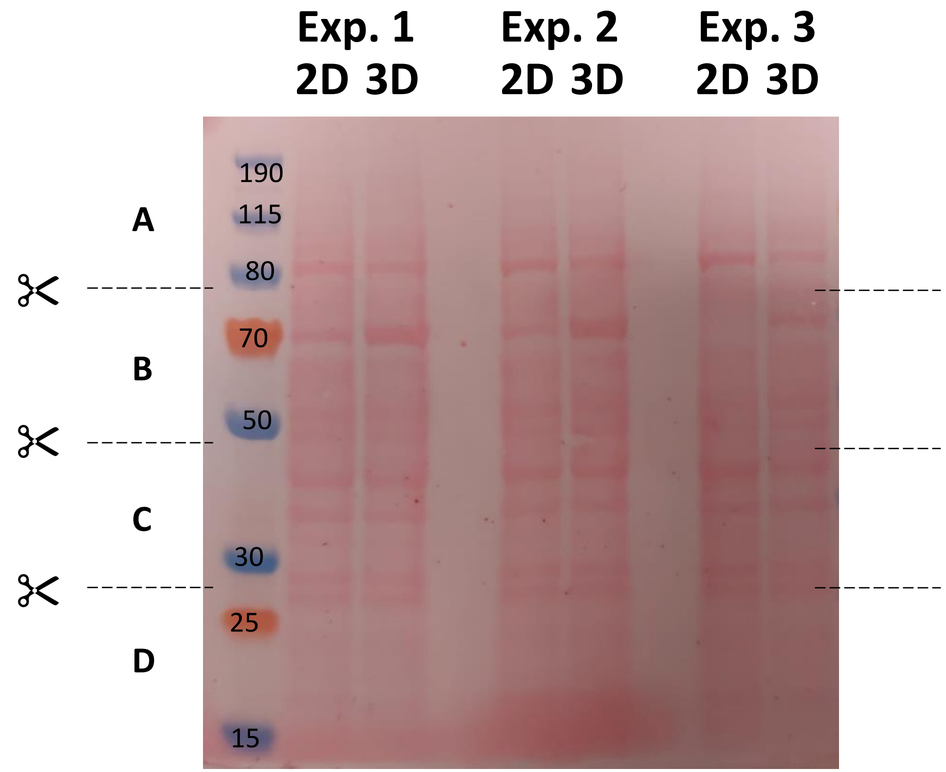

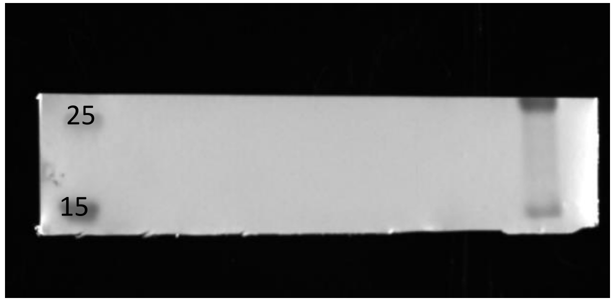

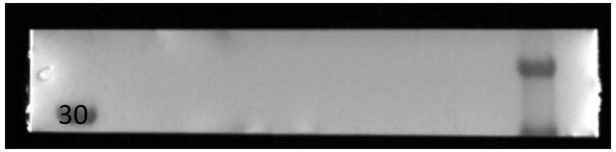

Part D_GPX4 (Molecular Weight: 20-22 KDa)

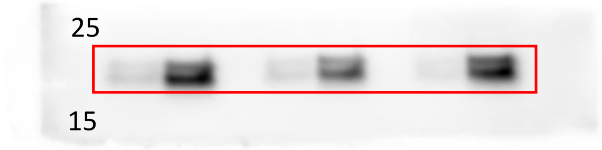

Merge

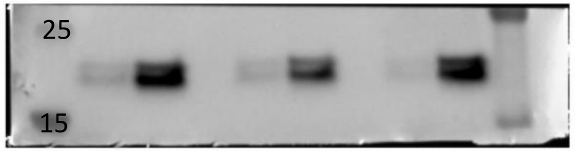

Part C_β-ACTIN (Molecular Weight: 42 KDa)

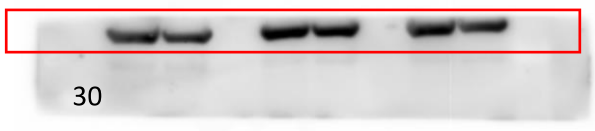

Merge

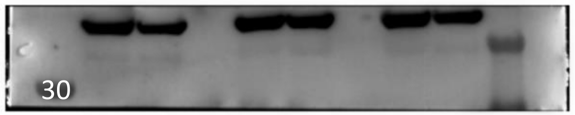

Relative to Figure 3 B

PUC30

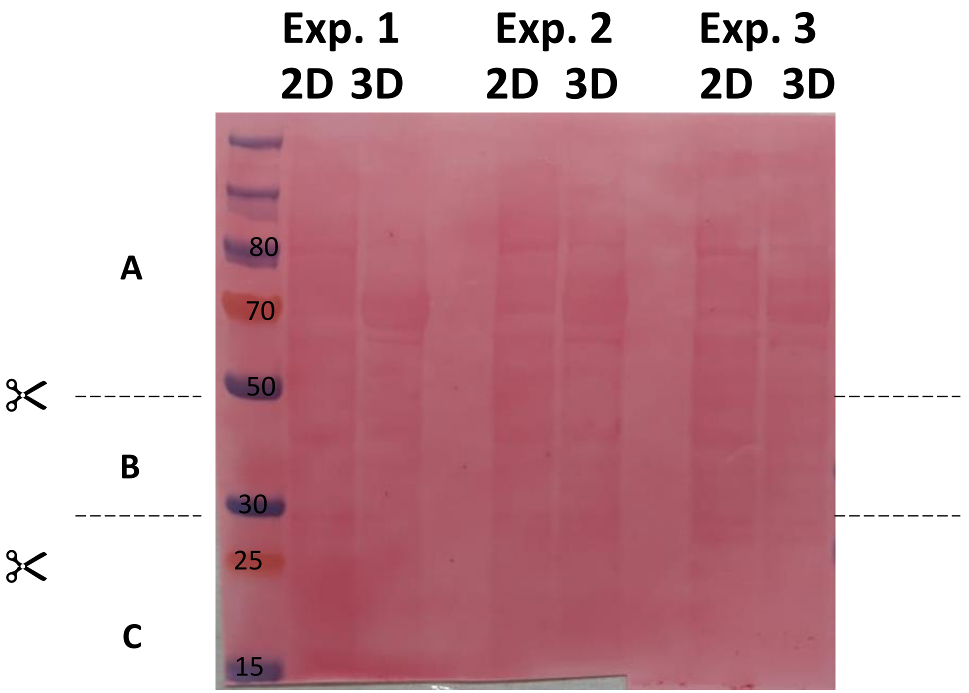

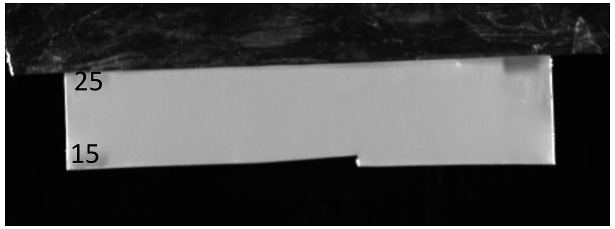

Part D_GPX4 (Molecular Weight: 20-22 KDa)

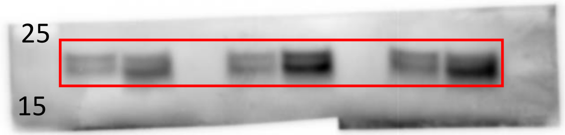

Merge

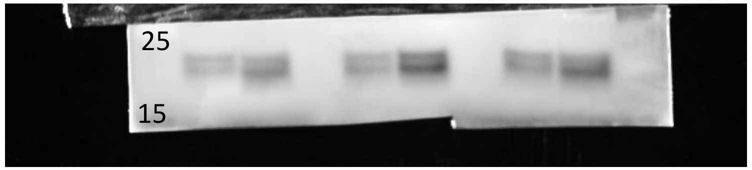

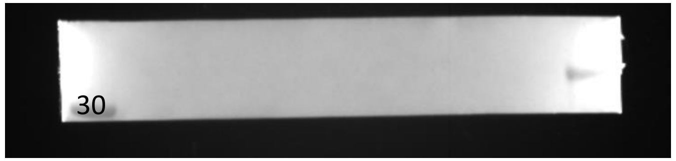

Part C_β-ACTIN (Molecular Weight: 42 KDa)

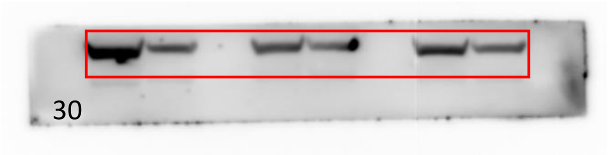

Merge

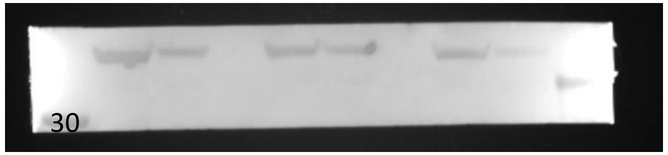

Relative to Figure 3 B

PUC36

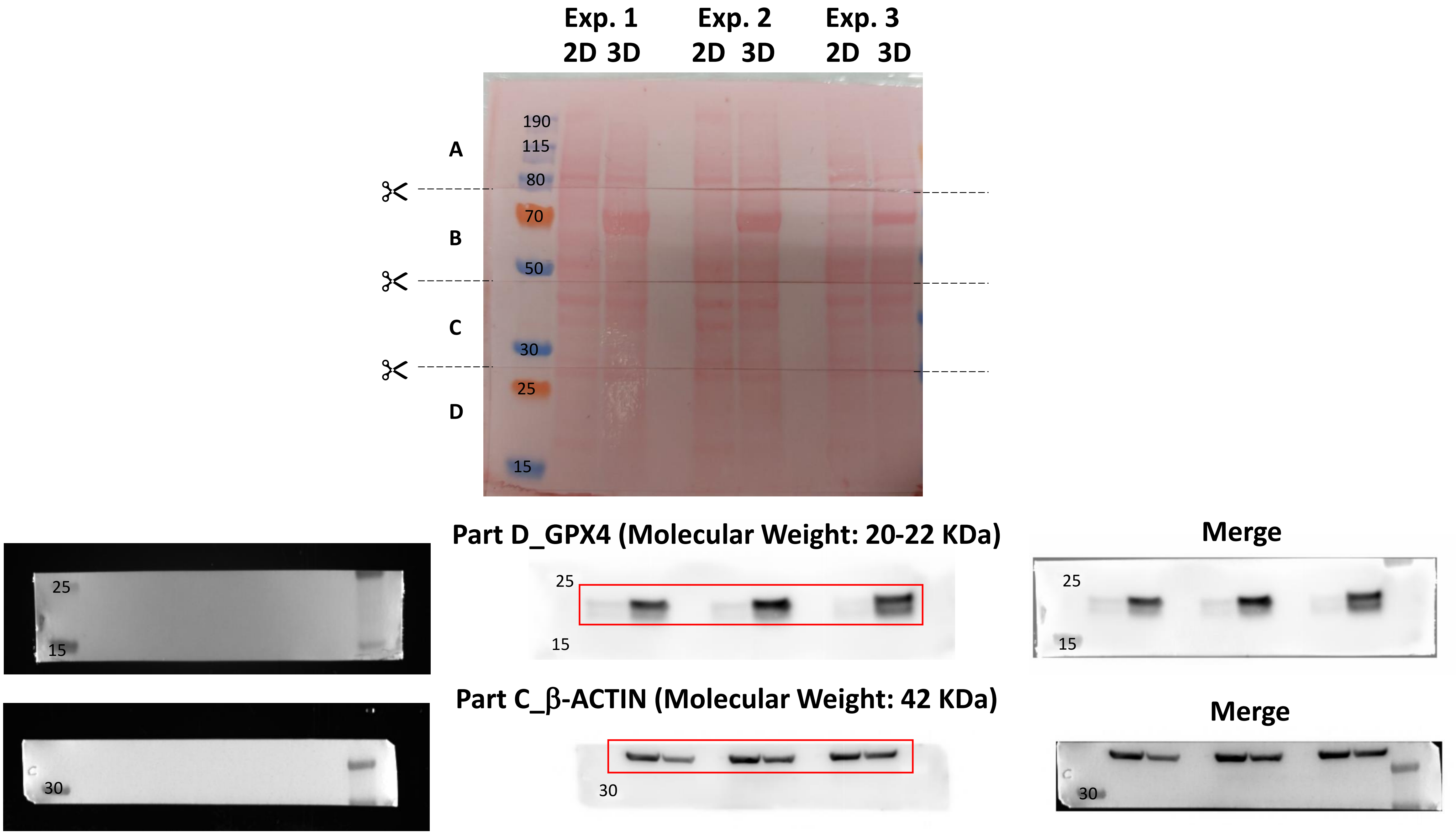

Relative to Figure 3 B

PUC37

Exp. 1 2D 3D | Exp. 2 2D 3D | Exp. 3 2D 3D

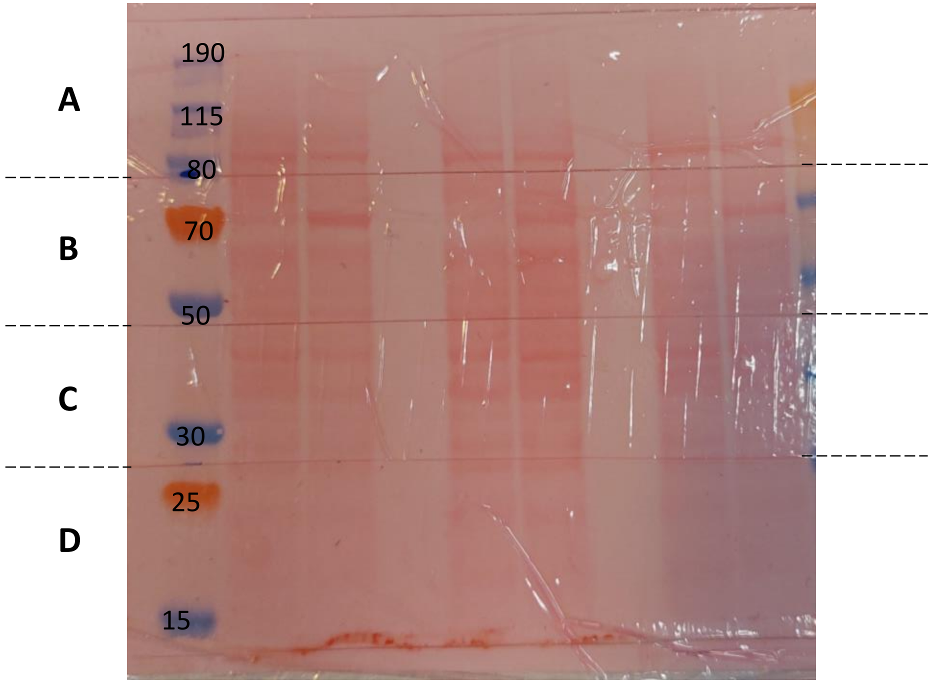

Part D_GPX4 (Molecular Weight: 20-22 KDa)

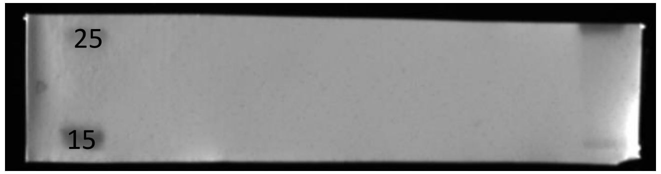

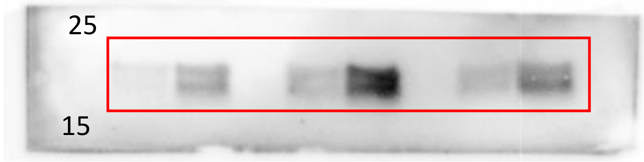

Merge

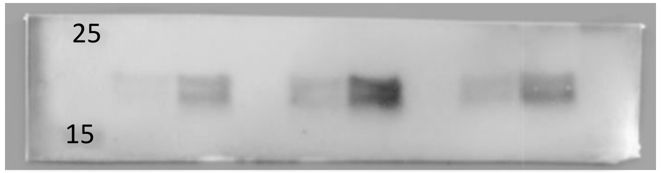

Part C_β-ACTIN (Molecular Weight: 42 KDa)

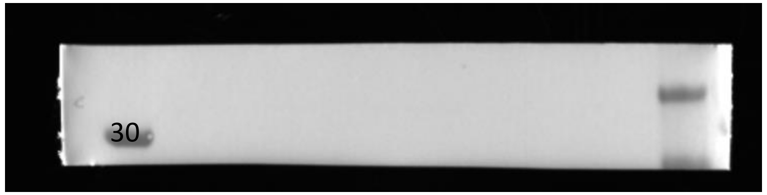

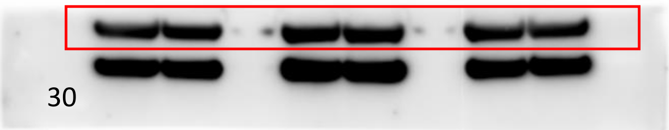

Merge

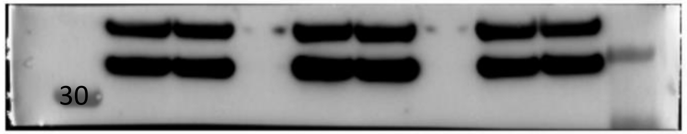

Relative to Figure 3 D

BBIRE-T248

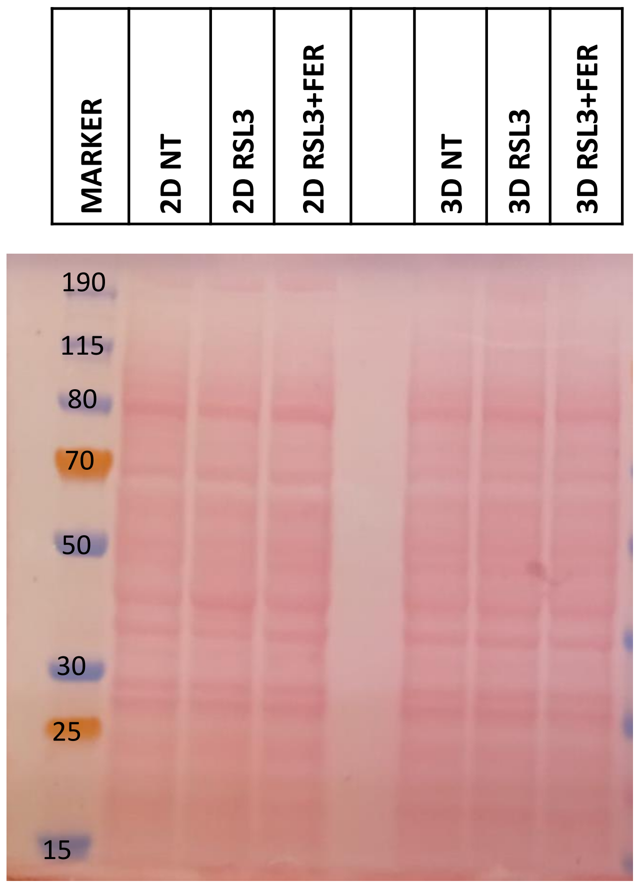

Relative to Figure 3 D

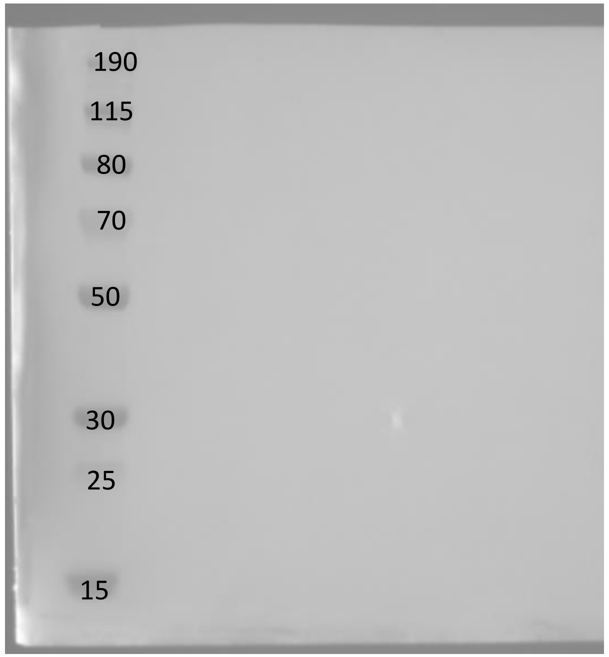

GPX4 (Molecular Weight: 20-22 KDa)

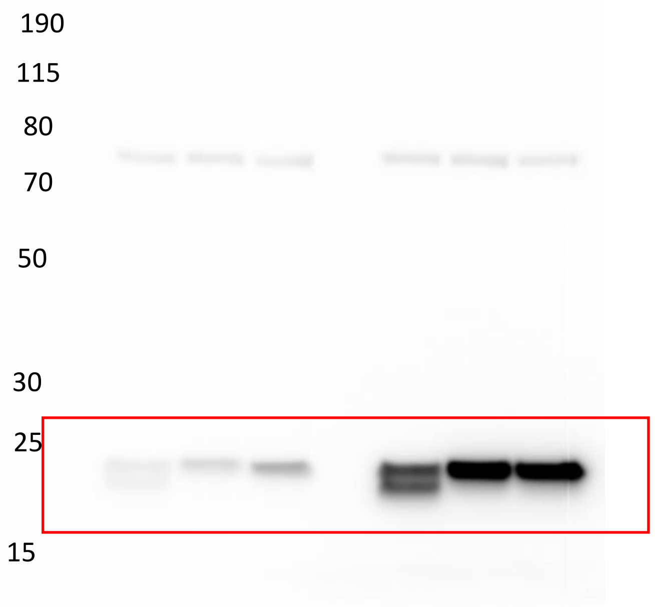

Merge

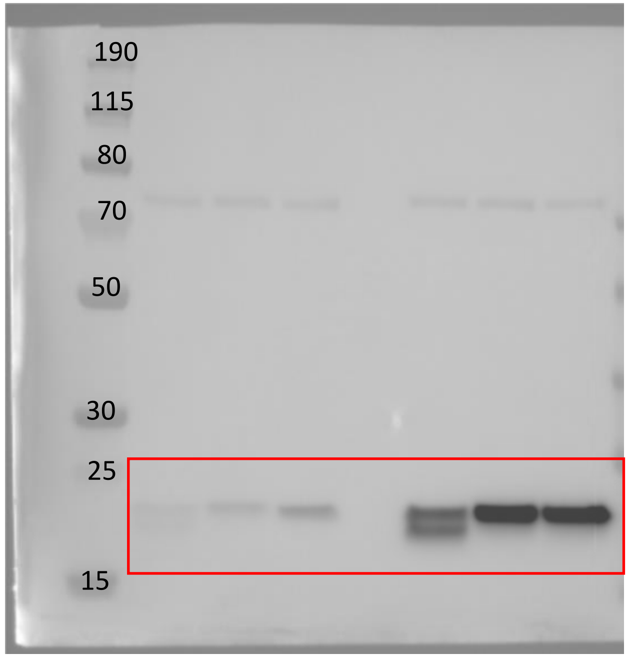

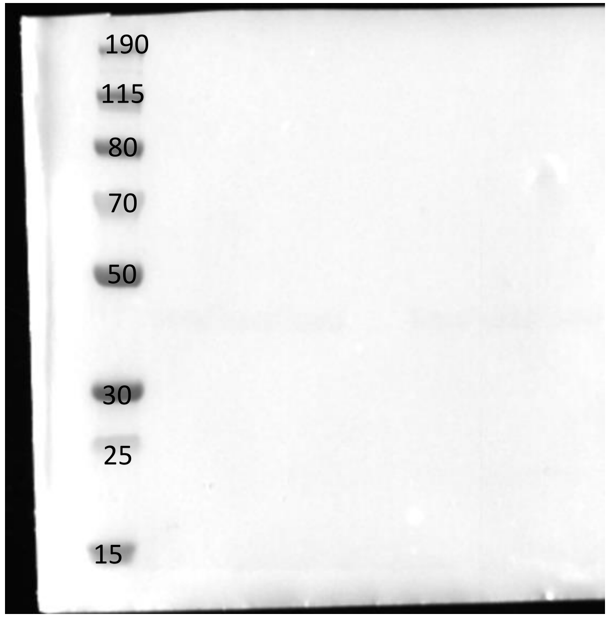

β-ACTIN (Molecular Weight: 42 KDa)

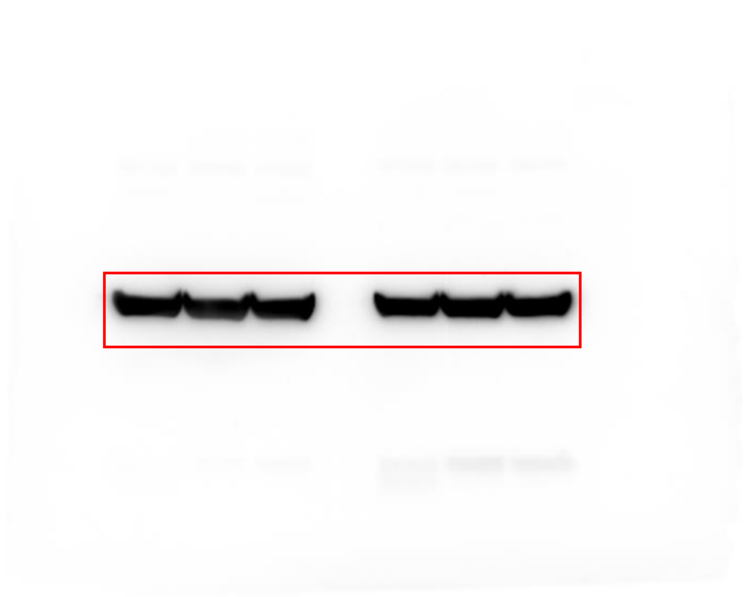

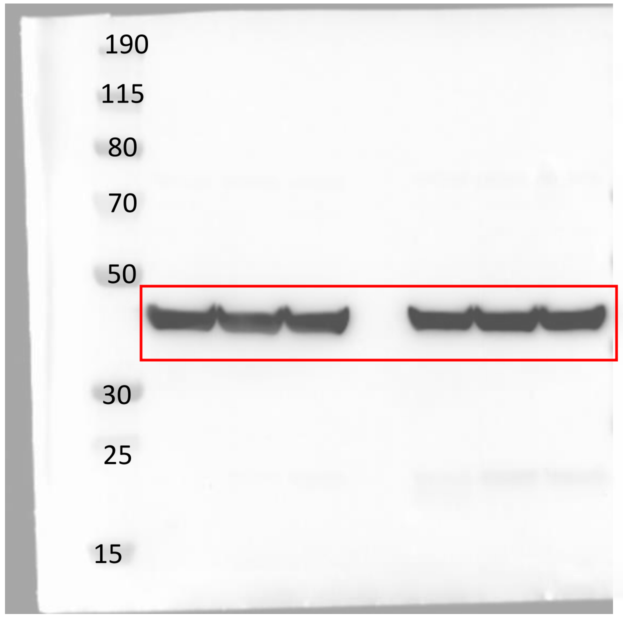

Supplement: Supplementary file 7 — Original western blots [file 41419_2024_6667_MOESM7_ESM.pdf]
